# Supplementary material for: SAFARI: Pangenome Alignment of Ancient DNA Using Purine/Pyrimidine Encodings
Source: bioRxiv. 2024 Oct 8:2024.08.12.607489. Preprint. [Version 2] doi: 10.1101/2024.08.12.607489 (PMC11482861; doi:10.1101/2024.08.12.607489)
Supplement: Supplement 1 [file media-1.pdf]

Supplementary Material for: `SAFARI`: Pangenome Alignment of Ancient DNA Using Purine/Pyrimidine Encodings

Joshua Rubin[1]*, Jan van Waaij[1], Louis Kraft[1], Jouni Sirén[2], Peter Wad Sackett[1], Gabriel Renaud[1]

October 8, 2024

1 Department of Health Technology, Section for Bioinformatics, Technical University of Denmark, Kongens Lyngby, Denmark
2 UC Santa Cruz Genomics Institute, Santa Cruz, USA

Contents

A Variation Graph Overview **3**

B Fragment Length Distribution Visualizations **3**

C Damage Rate Estimate Visualizations **4**

D Human and Archaic Hominin Panmitogenome Release **5**
D.1 Table of Archaic Hominin Sequences in the Human and Archaic Hominin Panmitogenome Release . . . 6
D.2 Phylogeny Inference and Ancestral State Reconstruction . . . 7
D.3 Panmitogenome Construction . . . 7
D.4 Improved Mapping of aDNA Reads from Archaic Hominins . . . 7
D.5 Effect of Misspecification of Damage Rate Matrices into `SAFARI` . . . 8
D.6 Surjection After Mapping to a Pangenome Can Benefit from Variation Even If the Linear Reference Is Identical . . . 9

E Inference Model Details **11**
E.1 User-defined Model Parameters in `SAFARI` . . . 11
E.2 Determination of Likelihood Function for Spurious Alignment . . . 11

F Experiment Details **13**
F.1 `HaploCart` Experiment . . . 13
F.2 Linear Aligner Benchmarking Experiment . . . 13
F.2.1 Exact Commands for the Linear Benchmarking Experiment . . . 14
F.2.2 (k, w) Indexing Parameter Pairs Evaluated . . . 15
F.2.3 Damage Rate Estimation Experiment . . . 15
F.3 Simulated Metagenomic Cave Environment Details . . . 16

*Email: jdru@dtu.dk

G Experiment Figures and Tables **17**
G.1 `HaploCart` Experiment 17
G.2 `euka` Experiment 26
G.2.1 Table of Detected Reads Per Taxon 26
G.2.2 Detected Reads Per Taxon by `SAFARI` Posterior Threshold 26
G.3 Linear Alignment Experiment 28
G.3.1 Selected Alignment Statistics 28
G.3.2 Sensitivity vs. Specificity Plots 29
G.3.3 Median F_1 Scores for Pangenome Aligners Using Default Indexing Parameter Values, Chagyrskaya Cave Distribution 31
G.3.4 Median F_1 Scores for Pangenome Aligners Using Sensitivity-optimized Indexing Parameter Values 32
G.3.5 Differences in Median Sensitivity/Specificity Between Pangenome Aligners By Indexing Parameter Values 33
G.3.6 Median F_1 Scores Across All Tools Using Chagyrskaya Cave Fragment Length Distribution 36
G.3.7 Raw Counts for Pangenome Aligners on Chagyrskaya Cave Fragment Length Distribution Dataset 36
G.3.8 Damage Rate Estimation 38

H Pseudocode **42**
H.1 Converting Kmer Keys to RYmer Keys 42
H.2 Filtering RYmers 42

I Experiment Memory and Runtime **43**
I.1 `HaploCart` and `euka` 43
I.2 Direct Comparison (`SAFARI` vs. `vg giraffe`) 43
I.3 Runtime as a Function of Minimizer/RYmer Index Values 43

J Experiment Data Details **44**
J.1 Nucleotide Misincorporation Matrices for Simulated Data 44
J.2 Downsampled Ancient BAM Files for `HaploCart` Experiment 47
J.3 Simulated Metagenomic Environment for `euka` Experiment 47

A Variation Graph Overview

For a good high-level overview of the variation graph data structure in general please see the Supplementary Material from [33], Section 1.

B Fragment Length Distribution Visualizations

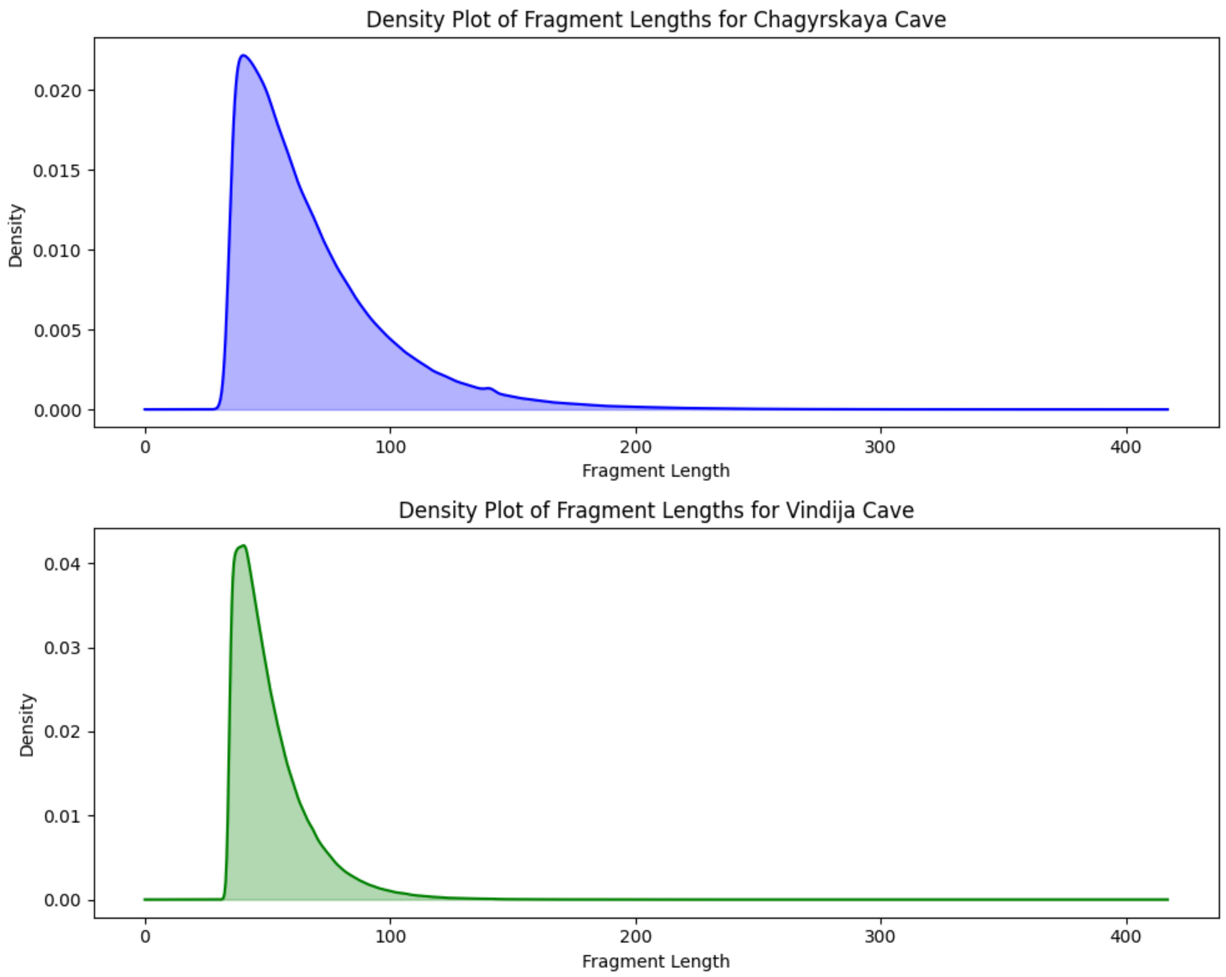

Figure 1: Fragment length distribution for the Chagyrskaya Cave [**TOP**] and Vindija Cave [**BOTTOM**] datasets, respectively.

C Damage Rate Estimate Visualizations

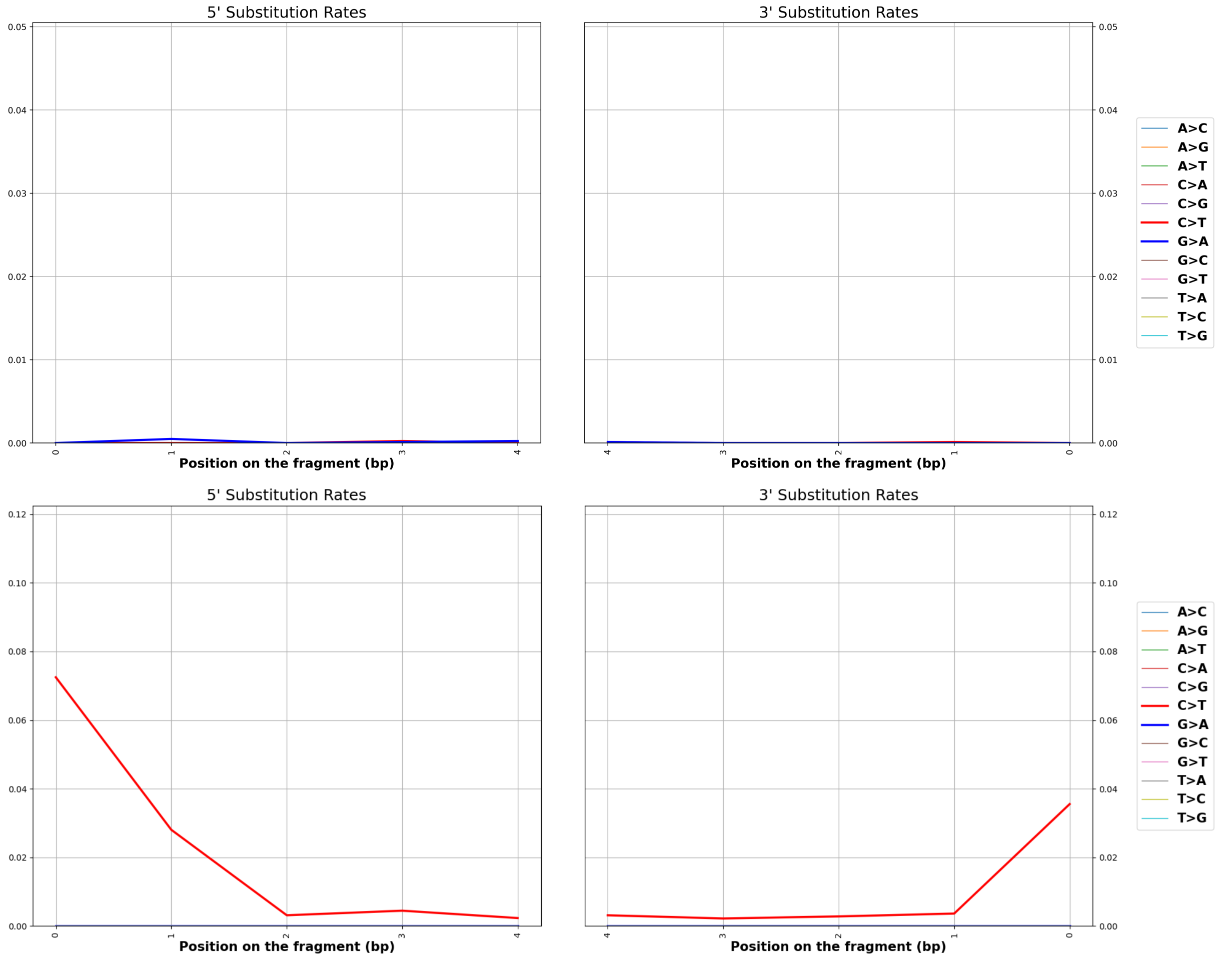

Figure 2: Examples of estimated damage rates for zero damage and single-stranded damage patterns used in our linear alignment experiments. Note that these BAM files were aligned with `SAFARI` but the corresponding BAM files aligned with `vg giraffe` are so similar that it would be difficult to distinguish.

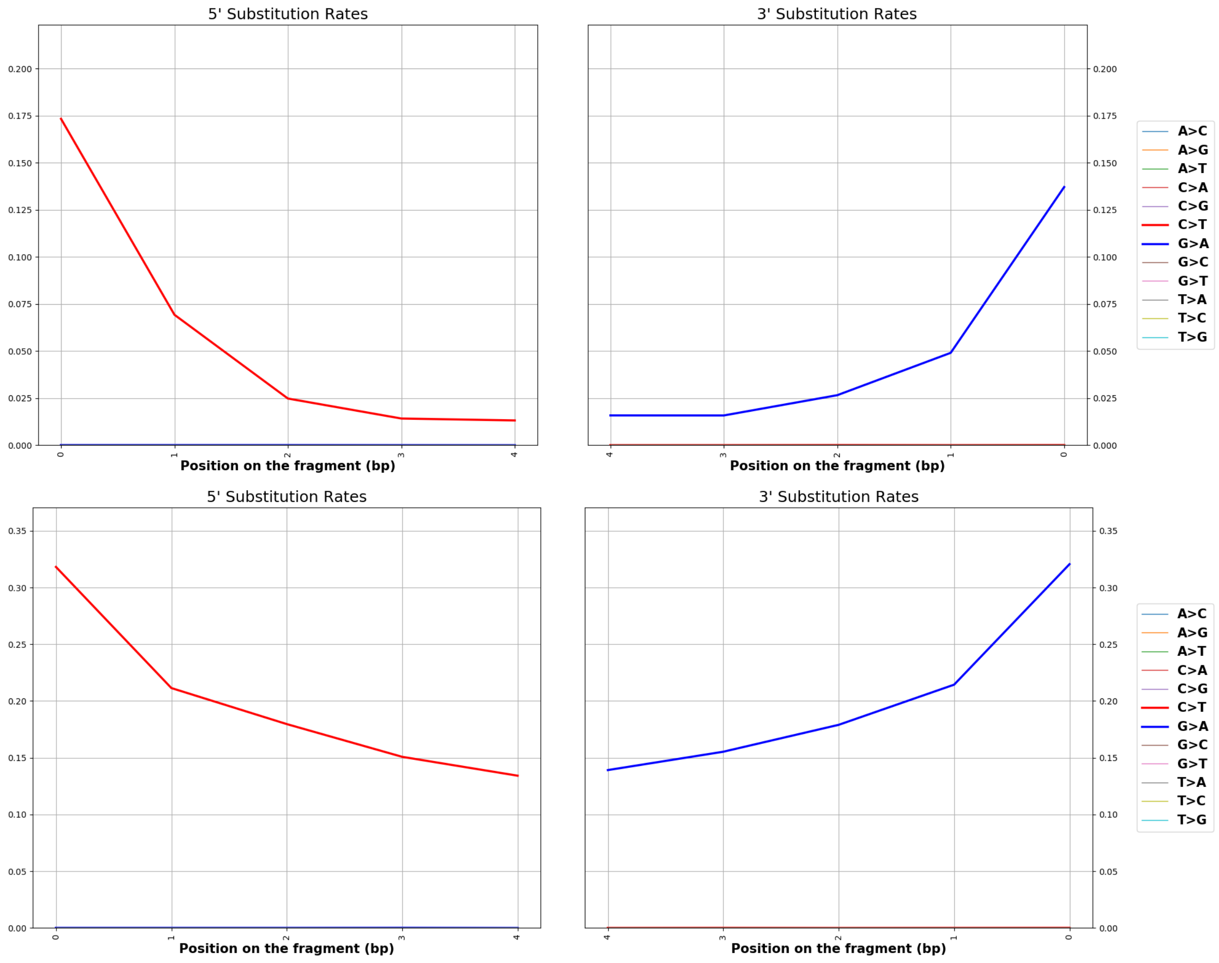

Figure 2: [**CONTINUED**] Examples of estimated damage rates for medium-level and high-level double-stranded damage patterns used in our linear alignment experiments.

D Human and Archaic Hominin Panmitogenome Release

As part of this paper we are releasing a curated pangenome reference graph for human and archaic hominin mitochondria. We hope this will help some researchers studying relevant samples to make use of our aDNA-aware aligner. The graph contains an update to our `HaploCart` graph with 6,401 human haplogroup sequences (an 18% increase from Phylotree Build 17, based on [6]). The increased resolution will better represent extant human mtDNA haplotype diversity. In addition there are archaic hominin sequences as well as ancestrally reconstructed states in order to better capture the true variation in hominin mitodiversity even for variation which no longer exists. In all the graph comprises 12,852 human and archaic hominin mitogenomes, to the best of our knowledge the largest panmitogenome ever published. These files are hosted in our in-house FTP server. In brief, the files can be retrieved via the Linux command

```
wget -nc -l1 --recursive --no-parent -P .
ftp://ftp.healthtech.dtu.dk:/public/SAFARI_graph/
```

Further details about the graph are provided in the subsections below.

D.1 Table of Archaic Hominin Sequences in the Human and Archaic Hominin Panmitogenome Release

Below we list all archaic hominin sequences used in the newly released panmitogenome as part of this paper.

Table 1: Comprehensive list of archaic hominin genomes used in the newly released panmitogenome.

Accession	Description
MT576650.1	Homo sapiens neanderthalensis isolate Denisova17 mitochondrion, complete genome
MT921957.1	Homo sapiens neanderthalensis mitochondrion, complete genome
KU131206.2	Homo sapiens neanderthalensis isolate DC1227 mitochondrion, complete genome
MG025536.1	Homo sapiens neanderthalensis isolate Les Cottes Z4-1514 mitochondrion, complete genome
KX198085.1	Homo sapiens neanderthalensis isolate GoyetQ374a-1 mitochondrion, complete genome
KX198086.1	Homo sapiens neanderthalensis isolate GoyetQ305-7 mitochondrion, complete genome
MG025540.1	Homo sapiens neanderthalensis isolate Goyet Q56-1 mitochondrion, complete genome
MG025538.1	Homo sapiens neanderthalensis isolate Spy 94a mitochondrion, complete genome
MG025539.1	Homo sapiens neanderthalensis isolate Vindija 87 mitochondrion, complete genome
NC_011137.1	Homo sapiens neanderthalensis mitochondrion, complete genome
KX198088.1	Homo sapiens neanderthalensis isolate GoyetQ57-2 mitochondrion, complete genome
MT677921.1	Homo sapiens neanderthalensis mitochondrion, complete genome
MG025537.1	Homo sapiens neanderthalensis isolate Mezmaiskaya 2 mitochondrion, complete genome
KX198087.1	Homo sapiens neanderthalensis isolate GoyetQ305-4 mitochondrion, complete genome
MK388903.1	Homo sapiens neanderthalensis isolate Chagyrskaya8 mitochondrion, complete genome
MT795654.1	Homo sapiens neanderthalensis isolate Stajnia S5000 mitochondrion, complete genome
OM062614.1	Homo sapiens neanderthalensis isolate Mezmaiskaya 3 mitochondrion, complete genome
MK033602.1	Homo sapiens neanderthalensis isolate Denisova15 mitochondrion, complete genome
MK123269.1	Homo sapiens neanderthalensis isolate Scladina I-4A mitochondrion, complete genome
KY751400.2	Homo sapiens neanderthalensis mitochondrion, complete genome
NC_023100.1	Homo heidelbergensis mitochondrion, complete genome
KT780370.1	Homo sapiens subsp. 'Denisova' isolate Denisova8 mitochondrion, complete genome
KX663333.1	Homo sapiens subsp. 'Denisova' isolate Denisova2 mitochondrion, complete genome

Continued on next page

Accession	Description
MT576651.1	Homo sapiens subsp. ‘Denisova’ isolate Denisova19 mitochondrion, complete genome
MT576653.1	Homo sapiens subsp. ‘Denisova’ isolate Denisova21 mitochondrion, complete genome
MT576652.1	Homo sapiens subsp. ‘Denisova’ isolate Denisova20 mitochondrion, complete genome

D.2 Phylogeny Inference and Ancestral State Reconstruction

We used `IQ-TREE` [23] to infer the phylogeny including ancestral states from our database of 6,401 modern human mitogenomes plus archaic hominin sequences (see above subsection). The choice of tool was based on feasibility given the inordinately large tree size; `IQ-TREE` is especially good at optimizing memory consumption, and for this reason previous researchers building large human mitochondrial phylogenies have also made use of this tool [2]. The program infers ancestral states in conjunction with inference of the phylogeny using a maximum likelihood approach. We construct the tree under a HKY DNA substitution model which allows for variable base frequencies (all bases) and a variable transition/transversion ratio, both essential features when dealing with mtDNA [12]. Specifically, we used the command

```
nice -19 iqtree -s [INPUT FILE] -m HKY -nt AUTO -redo -pre tree_tmp/haps -asr
```

D.3 Panmitogenome Construction

The hominin panmitogenome was constructed with `PGGB` [7]. The exact command used was as follows:

```
nice -19 parallel -j 55 --bar "nice pggb -i [INPUT MULTIFASTA] -t 50 -o final_s -p 70
-s  -H 12810 -n 5000 -k 300 -G 3079,3559 -l 20" :::  1100
```

We then used the `vg mod` subcommand to impose a maximum sequence size on each node of six nucleobases, as we found this empirically to help improve results. The final graph has 15722 nodes, 21551 edges, and 12852 embedded haplotype paths, and forms a single circularized connected component to reflect the biology of the eukaryotic mitogenome.

D.4 Improved Mapping of aDNA Reads from Archaic Hominins

Figure 3 shows how using `SAFARI` in conjunction with the released human and archaic hominin panmitogenome translates into more alignments for archaic samples in the presence of damage. The Denisovan sample used here has NCBI accession KT780370.1. The high-damage profiles are taken from [8]. With the exception of the user-inputted damage profiles for `SAFARI`, for which the appropriate ATP2 profile matrices were passed in, default parameters were used throughout for both tools. The number of aligned reads for each `in silico` sample was determined via the command `vg stats -a`. The data is presented in tabular form in Table 2 below.

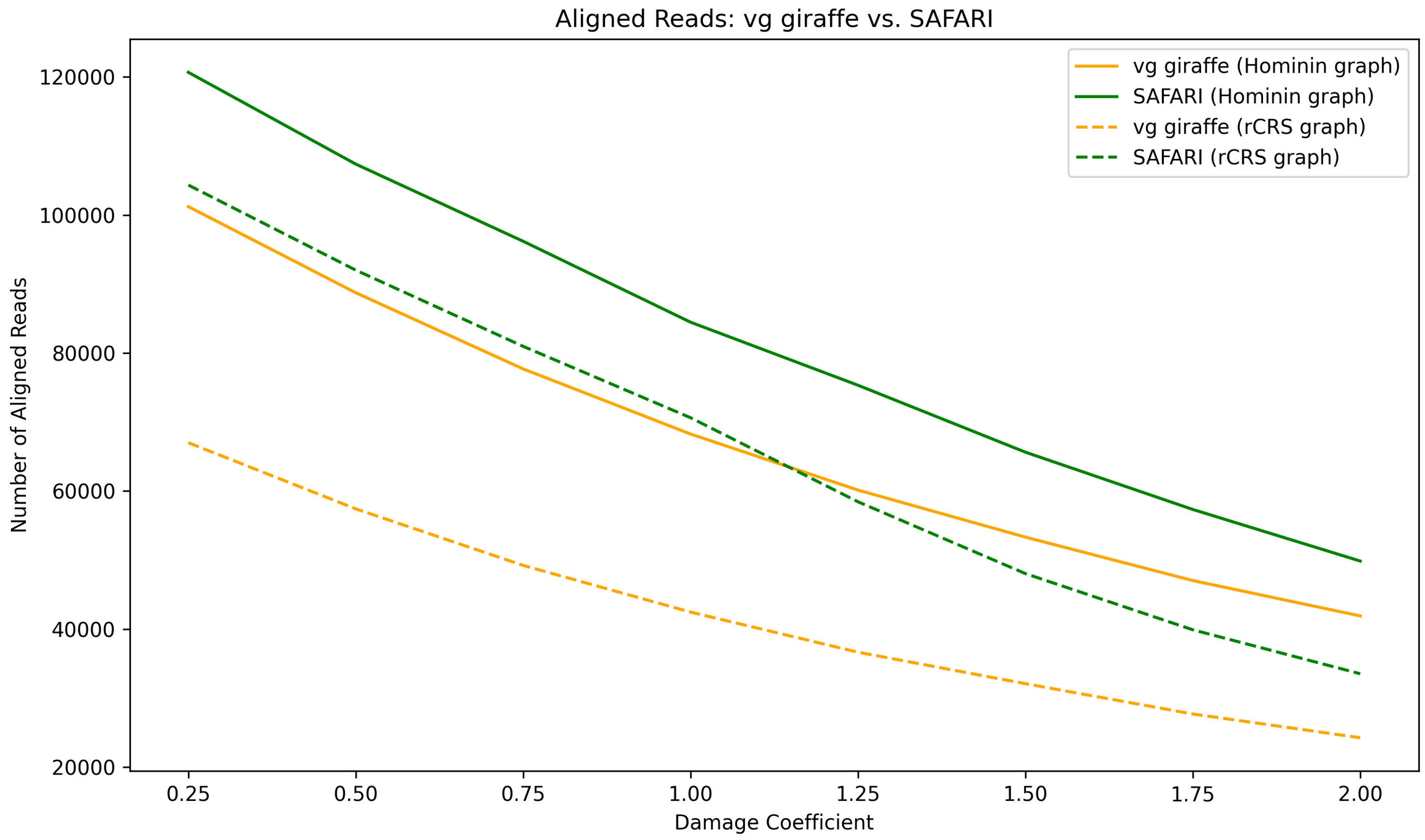

Figure 3: Number of aligned reads mapping with `vg giraffe` vs. `SAFARI` at various deamination rates for a Denisovan sample (generated *in silico*). The damage coefficient is a constant factor by which the deamination rates vary (in other words if the damage coefficient doubles then deamination events are twice as frequent). The dashed lines show data mapped to the linear reference graph containing only the rCRS reference genome, while the solid lines show data mapped to the full panmitogenome.

Table 2: Number of aligned reads mapping with `vg giraffe` vs. `SAFARI` at various deamination rates for a Denisovan sample (generated *in silico*).

Deamination factor	`vg giraffe` Hominin	`SAFARI` Hominin	`vg giraffe` rCRS	`SAFARI` rCRS
0.25	101,200	120,660	66,992	104,326
0.50	88,719	107,364	57,406	92,001
0.75	77,684	96,179	49,214	80,955
1.00	68,250	84,455	42,445	70,610
1.25	60,127	75,319	36,638	58,435
1.50	53,311	65,623	32,076	48,029
1.75	47,019	57,320	27,681	39,885
2.00	41,896	49,846	24,244	33,515

D.5 Effect of Misspecification of Damage Rate Matrices into `SAFARI`

As part of its model `SAFARI` requires the user to input estimated nucleotide misincorporation rates for the sample. It is therefore interesting to ask how sensitive the performance of the program is to these

estimates, and what the effect would be of underestimation or overestimation of damage in the input sample.

We generated 500,000 endogenous reads from a Denisovan sample (NCBI accession KT780370.1) [31]. These reads were contaminated (*in silico*) with 100,000 bacterial reads from the `RefSoil` database [5]. We then aligned the data with `SAFARI` at varying input estimated damage rate matrices. We did this by taking the ATP2_mt_1.00 rate matrices and multiplying the rates by a constant multiplicative factor which we term the damage coefficient. We vary the damage coefficient from 0.25 to 2.00, inclusive, at a step size of 0.25. This procedure was performed twice, once aligning to the full hominin panmitogenome and once to the single-haplotype graph comprising solely the rCRS haplotype H2a2a1.

We can see from Table 3 that, first and foremost, the program is not entirely insensitive to the damage rate estimates, in that varying the estimates will have an effect on the resulting alignments. However in general the program is quite robust to misspecifications of the damage rates. Furthermore, it is interesting to see that underestimation of damage seems to adversely affect performance more severely than overestimation of damage for both reference graphs, but this difference in sensitivity to error in the estimation is minor. Interestingly in both cases the number of resulting alignments as well as F_1 score peaks at a damage coefficient of 1.25, indicating that the `SAFARI` model is fairly well-calibrated, but could still benefit from minor adjustments in future work, as we would expect the performance to be optimal when the estimated rate matrices are closest to the true rate matrices.

Table 3: Alignment statistics and performance metrics for `SAFARI` mapping bacterially-contaminated Denisovan reads to both panmitogenomes at varying damage coefficient values (which affects the user-defined damage rate estimates passed into the program). All decimals are rounded to six figures.

Panmitogenome	Damage Coeff	TP	FP	TN	FN	Sensitivity	Specificity	F_1
Hominin	0.25	82890	245	24988	12	0.999855	0.990290	0.998452
Hominin	0.5	83023	248	24988	12	0.999855	0.990173	0.998437
Hominin	0.75	83191	248	24988	12	0.999856	0.990173	0.998440
Hominin	1.0	83333	248	24988	12	0.999856	0.990173	0.998442
Hominin	1.25	83482	248	24988	12	0.999856	0.990173	0.998445
Hominin	1.5	83377	248	24988	12	0.999856	0.990173	0.998443
Hominin	1.75	83406	248	24988	12	0.999856	0.990173	0.998444
Hominin	2.0	83457	249	24988	12	0.999856	0.990134	0.998439
rCRS	0.25	75892	210	24998	2	0.999974	0.991669	0.998605
rCRS	0.5	76224	210	24998	2	0.999974	0.991669	0.998611
rCRS	0.75	76457	210	24998	2	0.999974	0.991669	0.998616
rCRS	1.0	76707	210	24998	2	0.999974	0.991669	0.998620
rCRS	1.25	76961	210	24998	2	0.999974	0.991669	0.998625
rCRS	1.5	76878	210	24998	2	0.999974	0.991669	0.998623
rCRS	1.75	76921	210	24998	2	0.999974	0.991669	0.998624
rCRS	2.0	76921	210	24998	2	0.999974	0.991669	0.998624

D.6 Surjection After Mapping to a Pangenome Can Benefit from Variation Even If the Linear Reference Is Identical

Many ancient DNA researchers are accustomed to working with NGS pipelines which make use of a linear reference genome for alignment, and do processing on BAM files. Such researchers may well wonder if there is much point in mapping to a graph if they will need to surject alignments back to their reference haplotype downstream anyway, or whether all advantages accrued by the pangenome will be nullified after collapsing all the variation in the reference.

To test this we ran a quick experiment to show that indeed performance can improve by mapping to the full graph first and then surjecting afterwords. We took the same bacterially-contaminated Denisovan sample as in Section D.5 above. This sample was mapped by both aligners under identical parameters,

with the ATP2_mt_1.00 profiles being input into `SAFARI`. The mapping was performed twice, once to the single-haplotype rCRS reference graph, and then again to the full hominin panmitogenome, followed by surjection to the rCRS reference (H2a2a1).

Table 4: Comparison of performance metrics between aligning to the full hominin panmitogenome first and then surjecting onto the rCRS haplotype **[LEFT]** and directly aligning to the rCRS-only pangenome **[RIGHT]**. The former strategy can improve sensitivity, which is often critical for aDNA studies.

**Metric**	**Full Panmitogenome**	**rCRS Graph**
Corrected Alignments	87398	76707
Unmapped Bacteria	24988	24998
Mapped Bacteria	12	2
Mismapped Alignments	258	210

We see in Table 4 that considerably more reads can be aligned by first aligning to the full graph and then surjecting to the linear reference, despite the fact that the linear reference haplotype is very divergent from the true underlying haplotype in the sample. This is because if the panmitogenome contains reference genomes less divergent from the sample than the linear reference, it will store kmers in the index from those more similar genomes and extend and produce graphical alignments based on information from those more similar genomes. Then when surjection happens there will be mismatches etc. due to dissimilarity between the sample and the linear reference being surjected onto, but the alignments will still be produced, unlike if mapping directly where more alignments may fail either at the seed matching stage or the extension stage.

Although this procedure of pangenome mapping followed by surjection does lead to an increase in false positive mappings this issue is largely mitigated by the Bayesian filter in `SAFARI`.

E Inference Model Details

E.1 User-defined Model Parameters in SAFARI

We briefly discuss here some advice and general comments about user-defined parameters in our model. There are three user-defined parameters for SAFARI, namely the estimated nucleotide misincorporation matrices, the prior on a correct alignment of a base, and the posterior threshold. A discussion about the former (the estimated rate matrices) can be found in Section D.5.

The prior probability that a user should have on the likelihood that an arbitrary seed from their ancient sample will correctly align to the reference, conditionalized on a match of that seed to some indexed RYmer in the RYmer index (the flag "-V" in SAFARI) is heavily dependent on the nature of the sample. The degree of preservation, depth and breadth of coverage, and the specific methods used for DNA extraction, possible treatment, and sequencing are all factors which would have a substantial impact on this value. The user is therefore in a better position to provide a more informative prior than we are. Accordingly, we allow her to provide this information as a command-line argument. Similarly, the user can also control the posterior threshold (the flag "-j") to be able to adjust the sensitivity/specificity tradeoff to her requirements.

Unfortunately, even if the user has a good understanding of the sample in question, the choice of these parameters may still be difficult to fine-tune. This is because the probability will be very much dependent on the pangenome reference being used, the indexing parameters, and the nature of the sample, and future work is required to better understand and develop a model for this. Nonetheless the user may be able to use something like a grid search to identify optimal parameters for their sample, or perhaps best practices will be decided over time, similar to how optimal parameter settings for standard linear alignment tools for aDNA samples have reached broad consensus in the community.

E.2 Determination of Likelihood Function for Spurious Alignment

The question of what likelihood function to assign to model 2 (i.e. a spurious seed hit to the RYmer index) is a difficult one because in theory it will depend heavily on factors such as contamination rate and composition, which cannot be known *a priori*. In order to obtain a somewhat reasonable distribution, therefore, we generated a basic *in silico* simulation in Python to understand at a very coarse-grained level how the number of potentially deaminated mismatches between the seed and the candidate RYmer hit varies with the choice of kmer size k. Here we describe how we determined our likelihood function for a spurious alignment, conditionalized on the fact that there is a match in RYmer space between a seed and a hit.

We used the standard human mitochondrial reference, called the revised Cambridge Reference Sequence (rCRS), as our reference genome [1]. We indexed the minimizers and RYmers in the reference (https://github.com/JoshuaDanielRubin/SAFARI_paper_data/blob/main/spurious_model/main.py). The bacterial contamination FASTA file (https://github.com/JoshuaDanielRubin/SAFARI_data/blob/main/spurious_model/refSoilSmall.fa) comprising twenty bacterial genomes was obtained via accession numbers from the RefSoil database [5]. The mean genome length for the original file of 766 reference sequences was 4,396,003.8 bp. For reasons of space we used only the first twenty reference sequences to infer our spurious alignment model parameters.

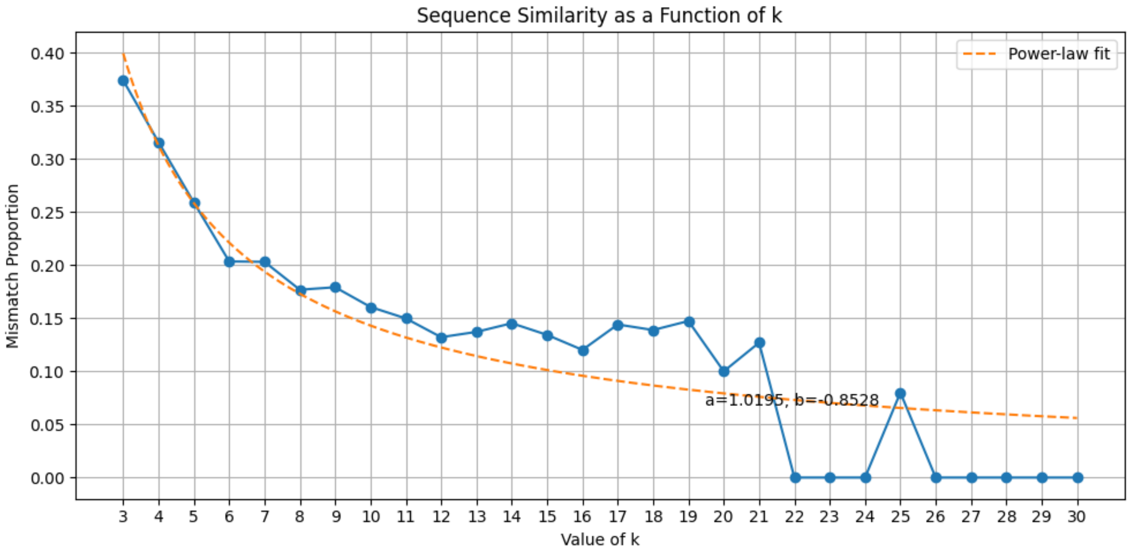

Figure 4: *in silico* `Python` simulations of aDNA RYmers and minimizers at varying values of k. The distribution roughly obeys a power law behavior which plateaus at high k values.

We emphasize that this likelihood function is very coarse-grained due to aleatoric uncertainties about the input sample which cannot in principle be known to us at runtime. However, we can confirm that this model is considerably more accurate in practice than a model assuming random DNA of equal base frequency for both the reference and the input data (where each base has probability 0.5 of mismatching in kmer space).

F Experiment Details

F.1 HaploCart Experiment

The script for downloading twenty ENA samples for the HaploCart experiment can be found at https://github.com/JoshuaDanielRubin/SAFARI_paper_data/blob/main/haplocart_ancient/download.sh. The script for downsampling samples can be found at https://github.com/JoshuaDanielRubin/SAFARI_paper_data/blob/main/haplocart_ancient/downsample.sh.

Note as well that for this experiment, we run with the -np flag, i.e. forgoing the phylogeny-aware confidence estimation as it is not relevant. We use the high-level damage matrices described in Section J.1 as input estimates.

F.2 Linear Aligner Benchmarking Experiment

Samples for the linear aligner benchmarking experiment were generated using the workflow manager Snakemake [13]. The Snakefile can be found at https://github.com/JoshuaDanielRubin/SAFARI_paper_data/blob/main/linear_experiment/human_mito/Snakefile.

In addition to our primary goal of comparing standard vg giraffe to SAFARI we also wanted to understand how well these tools perform with respect to commonly-used linear alignment tools. We therefore benchmarked against SHRiMP [30], BWA aln (both standard and ancient-specific parameters) [18, 21], BWA-MEM [16], BBMap [4], and Bowtie2 [14]. Though it is seldom used in the aDNA community and no longer maintained, SHRiMP uses a spaced kmer scheme which allows more tolerance of mismatches than standard kmer indexing systems, and we have internally found it to be highly effective for aligning aDNA samples [30].

Our reference genome was the rCRS (https://github.com/JoshuaDanielRubin/SAFARI_paper_data/blob/main/linear_experiment/human_mito/simulations/gen_0.fa). We stratified by four separate damage matrices. Three represent zero damage, medium-level damage, and high-level damage rates (i.e. nucleotide misincorporation patterns) for double-stranded DNA libraries. The fourth represents damage rates for single-stranded (otherwise know as Santa Cruz) libraries, which have been shown to be optimal in many cases for aDNA samples [10]. Further information about these matrices is provided in Section J.1.

We generated 300,000 circular (i.e. the end wraps around to the beginning, reflecting the circularity of mtDNA) mitochondrial fragments and 1,000 NuMT fragments using the fragSim subcommand of gargammel [32, 11, 28]. The fragment length distribution passed into fragSim via the -s flag was from either the empirically derived distribution from Vindija Cave, or that from Chagyrskaya Cave, depending on which dataset was being generated [20, 25]. aDNA damage was simulated using the deamSim subcommand of gargammel [28].

NuMTs are nuclear pseudogenes originating from the mitochondria which often mismap to the mitochondria [35]. They are found at low abundance relative to bona fide mtDNA, but in order to be extra charitable in our benchmarking and to ensure that our RYmer correction procedure is relatively robust to a high amount of false positives the proportion of NuMTs in our samples was well above the biological expectation. These fragments were contaminated *in silico* with 100,000 bacterial fragments from a *Campylobacter col* reference genome (NCBI accession NC_022660.1) [3] retrieved from the RefSoil database discussed in Section E.2. Fragment lengths for both the endogenous and contaminant material obeyed an empirically derived distribution from a high-coverage 80,000-year-old Neanderthal sample from Chagyrskaya Cave [20]. Samples were subsampled at eighteen subsampling rates (0.1 to 0.95 inclusive, step size 0.05) using seqtk [17]. Adapter sequences were added using the adptSim subcommand of gargammel. Illumina HiSeq 2500 short reads were then simulated using ART [9] at a fixed read length of 125 bp. Adapter sequences were trimmed and these reads were merged using leeHom with the --ancientdna flag enabled [27]. Exact command and parameter settings can be found in the Snakefile. A total of 568 BAM files were produced by each of the eight alignment tools under evaluation, yielding a total sample size of 4544 alignment files.

F.2.1 Exact Commands for the Linear Benchmarking Experiment

For `BWA aln` we follow [21] in setting the aDNA-specific parameters. For `BWA-MEM` we follow [34]. For `Bowtie2` we follow [14]. Parameters for the other tools were decided upon from prior experience/experimentation.

Table 5: Exact commands used in the linear aligner benchmarking experiment.

Rule	Command
BWA-MEM	interleavefastq.sh {input_1} {input_2} {input_3} | bwa mem -r 2.5 -k 19 -p simulations/gen_0.fa /dev/stdin | samtools sort /dev/stdin > {output}
BWA aln	cat <(bwa aln simulations/gen_0.fa {input_1} | bwa samse simulations/gen_0.fa /dev/stdin {input_1}) <(bwa sampe simulations/gen_0.fa <(bwa aln simulations/gen_0.fa {input_2}) <(bwa aln simulations/gen_0.fa {input_3}) {input_2} {input_3} | samtools view -S /dev/stdin) | samtools sort /dev/stdin > {output}
BWA aln (anc)	cat <(bwa aln -n 0.02 -o 2 -l 1024 simulations/gen_0.fa {input_1} | bwa samse simulations/gen_0.fa /dev/stdin {input_1}) <(bwa sampe simulations/gen_0.fa <(bwa aln -n 0.02 -o 2 -l 1024 simulations/gen_0.fa {input_2}) <(bwa aln -n 0.02 -o 2 -l 1024 simulations/gen_0.fa {input_3}) {input_2} {input_3} | samtools view -S /dev/stdin) | samtools sort /dev/stdin > {output}
Bowtie2	interleavefastq.sh {input_1} {input_2} {input_3} | bowtie2 --end-to-end --sensitive -x bowtie2index/gen_0.fa --interleaved /dev/stdin | samtools sort /dev/stdin > {output}
SHRiMP	cat <(gmapper -N 1 -o 1 --single-best-mapping --sam-unaligned --fastq --sam --no-qv-check --qv-offset 33 {input_1} simulations/gen_0.fa) <(gmapper -N 1 -o 1 --single-best-mapping --sam-unaligned --fastq --sam --no-qv-check --qv-offset 33 -1 {input_2} -2 {input_3} simulations/gen_0.fa | samtools view -S /dev/stdin) | samtools sort /dev/stdin > {output}
BBMap	cat <(bbmap.sh sam=1.3 qin=33 ref=simulations/gen_0.fa nodisk=t in={input.input_1} out=/dev/stdout) <(bbmap.sh sam=1.3 qin=33 ref=simulations/gen_0.fa nodisk=t in={input.input_2} in2={input.input_3} out=/dev/stdout | samtools view -S /dev/stdin) | samtools sort /dev/stdin > {output}
SAFARI	nice -19 vg safari -f /dev/stdin -m graph.min -d graph.dist -Z graph.giraffe.gbz --deam-3p [3p DEAM PROFILE] --deam-5p [5p DEAM PROFILE] -j 0.2 | vg surject -x graph.xg -P -F PATHS -b /dev/stdin | samtools sort /dev/stdin > {output}
vg giraffe	nice -19 interleavefastq.sh input_1} {input_2} {input_3} | vg giraffe -f /dev/stdin -m graph.min -d graph.dist -Z graph.giraffe.gbz | vg surject -x graph.xg -P -F PATHS -b /dev/stdin | samtools sort /dev/stdin > {output}

F.2.2 (k, w) Indexing Parameter Pairs Evaluated

In addition to the default indexing parameters we also aligned all samples under 25 other distinct indexing parameter pairs for both `vg giraffe` and `SAFARI` in order to understand how varying these parameter values affect the behavior of these tools. Below (Table 6) is a table listing the parameter pairs that were evaluated.

Table 6: Indexing parameter pair values for `SAFARI` and `vg giraffe` evaluated in the linear alignment experiment.

k	w
10	10
10	15
10	2
10	5
10	7
10	8
11	6
11	7
12	3
12	4
12	5
12	6
12	7
15	10
15	15
15	5
18	8
20	10
20	15
20	2
20	5
29	11
30	10
30	15
30	2
30	5

F.2.3 Damage Rate Estimation Experiment

Populating the MD Field in `SAFARI` and `vg giraffe` Alignments It is a minor annoyance that natively `SAFARI` and `vg giraffe` surjected alignments in BAM file format do not populate the MD field required to represent mismatches between the read and the reference (although mismatches are directly reported in GAM file format). Therefore these fields were populated afterwards via the `samtools calmd` subcommand [19]. For the script that does this please see https://github.com/JoshuaDanielRubin/SAFARI_paper_data/blob/main/linear_experiment/human_mito/make_MD.sh.

Damage Profile Estimates with `bam2prof` `bam2prof` is a small script for estimating nucleotide misincorporation rates from BAM file input [26]. For the samples with damage patterns arising from a single-stranded library preparation we ran `bam2prof` with flags `-minl 20 -q -single -minq 0 -length 5`. For all other samples we run with the flags `-minl 20 -q -both -minq 0 -length 5`.

F.3 Simulated Metagenomic Cave Environment Details

For full details of the simulated metagenomic cave environment used in the `euka` experiment please see https://github.com/nicolaavogel/eukaPaperData/tree/master/CreateSimulatedEnv. There you will find the precise bacteria, fungi, archaea, and viruses included in the sample. A table of the present eukaryotic taxa is provided below. In total there were 1500000 reads in the full sample.

Bovidae
Calyptratae
Colubroidea
Cryptorhynchinae
Lampyridae
Lycosoidea
Mus
Myotis
Neognathae
Rattus
Sciurinae
Ursidae
Xerinae

Table 7: Eukaryotic taxa present in the simulated metagenomic environment.

G Experiment Figures and Tables

G.1 HaploCart Experiment

Table 8: Comparison of predicted human mtDNA haplogroup and number of processed reads between the original and modified HaploCart versions at four target mean coverage depths (0.25X, 0.5X, 1X, and 2X) over five replicates. Full coverage predictions for HaploCart and HaploGrep2, respectively, are provided in the third column. Across the board, the total number of processed reads increases. More importantly, the predictions are more accurate with respect to edit distance between predicted and ground truth haplogroup labels as described in the main article Section 3.2.

Sample	Rate	Full_Cov_Pred	Replicate	HG_corr	HG_uncorr	Reads_corr	Reads_uncorr
AED204	0.25X	X2b11	1	X1'2'3	B4a2	49	42
AED204	0.25X	X2b11	2	X2b1	X2b	64	52
AED204	0.25X	X2b11	3	X2	X2	54	48
AED204	0.25X	X2b11	4	N1a1b	X4	59	51
AED204	0.25X	X2b11	5	X2b12	X2b12	42	36
AED92b	0.25X	U4a1	1	U4a2d	U2b	45	36
AED92b	0.25X	U4a1	2	U4a1	U4a1	68	62
AED92b	0.25X	U4a1	3	U4a1	U4d1	58	49
AED92b	0.25X	U4a1	4	U4	U4	66	57
AED92b	0.25X	U4a1	5	U4a1	U4a1	65	60
Alh10	0.25X	I1	1	I1	I1	46	44
Alh10	0.25X	I1	2	H27a	B4c1a1a1	44	35
Alh10	0.25X	I1	3	A1	A1	42	38
Alh10	0.25X	I1	4	I6a	I6a	61	52
Alh10	0.25X	I1	5	I1	I1	39	36
DA100	0.25X	C4b1	1	C4	C4	41	39
DA100	0.25X	C4b1	2	C4	C4	54	51
DA100	0.25X	C4b1	3	C4b	C4b	36	35
DA100	0.25X	C4b1	4	C4	C4	43	40
DA100	0.25X	C4b1	5	C4b1	C4b1	34	28
DA101	0.25X	U5a1b1e	1	U5a	U5a	50	47
DA101	0.25X	U5a1b1e	2	U5a1b1	U5a1b1	49	46
DA101	0.25X	U5a1b1e	3	U5a	U5a	42	38
DA101	0.25X	U5a1b1e	4	U5a1b1e	U5a1b1e	48	45
DA101	0.25X	U5a1b1e	5	U6c	H2a1f2	43	41
DA15	0.25X	C4d	1	C1	C1	54	48
DA15	0.25X	C4d	2	C4e	C4e	55	49
DA15	0.25X	C4d	3	C4e	C4e	58	54
DA15	0.25X	C4d	4	C4d	C4d	61	56
DA15	0.25X	C4d	5	C4e	C4e	48	45
DA171	0.25X	H2a1	1	H3u	H1bd	57	50
DA171	0.25X	H2a1	2	M35a	M35a	59	54
DA171	0.25X	H2a1	3	U5a2e	U5a2e	61	53
DA171	0.25X	H2a1	4	H5p	H5p	55	47
DA171	0.25X	H2a1	5	B5	B4m	63	58
FN2	0.25X	H3	1	H24a1	H24a1	49	44
FN2	0.25X	H3	2	H1aw	L3	52	48
FN2	0.25X	H3	3	H56	H1	53	46
FN2	0.25X	H3	4	H27c	H27c	51	41

Continued on next page

Sample	Rate	Full_Cov_Pred	Replicate	HG_corr	HG_uncorr	Reads_corr	Reads_uncorr
FN2	0.25X	H3	5	H27	H10	32	25
I10899	0.25X	U5b	1	U5b1	U5b1	88	86
I10899	0.25X	U5b	2	U5a	U5a	92	91
I10899	0.25X	U5b	3	L3e2a	L3e2a	107	103
I10899	0.25X	U5b	4	U5a'b	U5a'b	82	78
I10899	0.25X	U5b	5	U5b1	U5b1	108	104
I11300	0.25X	J2a1a1	1	J2a1a1	J2a1a1	80	76
I11300	0.25X	J2a1a1	2	J2a1a1	J2a1a1	110	107
I11300	0.25X	J2a1a1	3	J2a1a1	J2a1a1	83	80
I11300	0.25X	J2a1a1	4	J2a1a1	J2a1a1	103	98
I11300	0.25X	J2a1a1	5	J2	J2	88	84
I7645	0.25X	R7b2	1	B4'5	B4'5	35	32
I7645	0.25X	R7b2	2	B2	B2	61	59
I7645	0.25X	R7b2	3	T2b22	T2b22	49	49
I7645	0.25X	R7b2	4	B2	B2	52	49
I7645	0.25X	R7b2	5	B4'5	B4'5	68	64
I7646	0.25X	H1e1c	1	H1bi	H1bi	68	66
I7646	0.25X	H1e1c	2	H1e1c	H1e1c	68	63
I7646	0.25X	H1e1c	3	J1d	J1d	61	54
I7646	0.25X	H1e1c	4	H1bi	H1bi	70	69
I7646	0.25X	H1e1c	5	H1	H1	78	72
I8132	0.25X	D1a2	1	U8a1b	U8a1b	49	47
I8132	0.25X	D1a2	2	J1c2f	J1c2f	42	40
I8132	0.25X	D1a2	3	B2	B2	40	39
I8132	0.25X	D1a2	4	H1aq	H1aq	51	48
I8132	0.25X	D1a2	5	H1aq	H1aq	48	48
I8569	0.25X	H1ah	1	H1	H1	64	64
I8569	0.25X	H1ah	2	B4	B4	69	66
I8569	0.25X	H1ah	3	B4'5	B4'5	65	65
I8569	0.25X	H1ah	4	B4	B4	71	68
I8569	0.25X	H1ah	5	H1	H1	63	62
NW54	0.25X	C4a1a1	1	C4b5	C1d1	56	46
NW54	0.25X	C4a1a1	2	C1b8a	C1b8a	45	38
NW54	0.25X	C4a1a1	3	C4d	C4d	59	47
NW54	0.25X	C4a1a1	4	C4a1a1	C4a1a1	65	57
NW54	0.25X	C4a1a1	5	C4a1a	M18	56	48
STR266b	0.25X	J1c5	1	F1	U4a2h1	34	29
STR266b	0.25X	J1c5	2	J1	J1	46	41
STR266b	0.25X	J1c5	3	U5b2a1b	U5b2a1b	44	40
STR266b	0.25X	J1c5	4	J1c9	J1c9	56	51
STR266b	0.25X	J1c5	5	J1c7	J1c7	59	53
STR393b	0.25X	H5a1	1	H2a1f	H2a1f	50	43
STR393b	0.25X	H5a1	2	H1c4a1	H44b	56	42
STR393b	0.25X	H5a1	3	H5s	H5s	54	41
STR393b	0.25X	H5a1	4	V25	V25	52	43
STR393b	0.25X	H5a1	5	H3w	H3w	55	44
STR486	0.25X	T2b	1	T2f5	T1	50	43
STR486	0.25X	T2b	2	T2	T2	50	41
STR486	0.25X	T2b	3	T1	T1	47	42
STR486	0.25X	T2b	4	T2	T2	56	49

Continued on next page

Sample	Rate	Full_Cov_Pred	Replicate	HG_corr	HG_uncorr	Reads_corr	Reads_uncorr
STR486	0.25X	T2b	5	T2b	T2b	56	45
STR491	0.25X	T2b	1	T2b	T2b	55	47
STR491	0.25X	T2b	2	T2b33	T2b33	42	37
STR491	0.25X	T2b	3	T2b	T2e1a1b1	52	44
STR491	0.25X	T2b	4	T2b	T1	44	36
STR491	0.25X	T2b	5	T2	T2	46	43
Vim2b	0.25X	H7	1	H1	H72	46	38
Vim2b	0.25X	H7	2	H2a1f1	H2a1f1	47	38
Vim2b	0.25X	H7	3	HV17	HV17	43	40
Vim2b	0.25X	H7	4	H1	H1	47	34
Vim2b	0.25X	H7	5	H1c7	H1c7	42	36
AED204	0.5X	X2b11	1	X2b	B4	99	81
AED204	0.5X	X2b11	2	X2b6	X2b6	104	88
AED204	0.5X	X2b11	3	X2b9	A2e	115	102
AED204	0.5X	X2b11	4	X2b4	X2b4	96	80
AED204	0.5X	X2b11	5	X2b	X2b	123	102
AED92b	0.5X	U4a1	1	U4a1	U4a1	116	103
AED92b	0.5X	U4a1	2	U4a1d	N22a	120	101
AED92b	0.5X	U4a1	3	U4a1	U4a1	115	101
AED92b	0.5X	U4a1	4	U4a1	U4a1	124	103
AED92b	0.5X	U4a1	5	U4a1	U4a1	144	128
Alh10	0.5X	I1	1	I1	I1	84	69
Alh10	0.5X	I1	2	I1	I1	91	72
Alh10	0.5X	I1	3	I1	I1	100	89
Alh10	0.5X	I1	4	I1c	I1	97	85
Alh10	0.5X	I1	5	I1	I1	84	76
DA100	0.5X	C4b1	1	C4b1	C4b1	100	91
DA100	0.5X	C4b1	2	C4b5	C4b5	90	87
DA100	0.5X	C4b1	3	C4	C4	76	69
DA100	0.5X	C4b1	4	C4b1	C4b1	92	84
DA100	0.5X	C4b1	5	C4	C4	75	68
DA101	0.5X	U5a1b1e	1	U5a1b1e	U5a1b1e	90	82
DA101	0.5X	U5a1b1e	2	U5a1b1e	U5a1b1e	110	103
DA101	0.5X	U5a1b1e	3	U5a2a1a	U5a2a1a	103	94
DA101	0.5X	U5a1b1e	4	U5a1b1e	U5a1b1e	103	97
DA101	0.5X	U5a1b1e	5	U5a1	U5a1	95	84
DA15	0.5X	C4d	1	C1	C1	110	98
DA15	0.5X	C4d	2	C1	C1	120	112
DA15	0.5X	C4d	3	C4	C4	120	109
DA15	0.5X	C4d	4	C4	C4	132	114
DA15	0.5X	C4d	5	C4d	C4d	98	86
DA171	0.5X	H2a1	1	H2a1	H2a1	116	102
DA171	0.5X	H2a1	2	H107	H1an2	118	111
DA171	0.5X	H2a1	3	H5p	H5p	113	98
DA171	0.5X	H2a1	4	H1an2	H1an2	104	96
DA171	0.5X	H2a1	5	H1an2	H1an2	114	104
FN2	0.5X	H3	1	H48	H48	114	89
FN2	0.5X	H3	2	H104	H104	117	95
FN2	0.5X	H3	3	H5a9	H5a9	104	90
FN2	0.5X	H3	4	H16e	H16e	116	88

Continued on next page

Sample	Rate	Full_Cov_Pred	Replicate	HG_corr	HG_uncorr	Reads_corr	Reads_uncorr
FN2	0.5X	H3	5	H3at1	H105	90	80
I10899	0.5X	U5b	1	U5b1	U5b1	210	207
I10899	0.5X	U5b	2	U5b1	U5b1	161	157
I10899	0.5X	U5b	3	U5b1	U5b1	201	197
I10899	0.5X	U5b	4	U5b1h	U5b1h	172	169
I10899	0.5X	U5b	5	U5b	U5b	172	169
I11300	0.5X	J2a1a1	1	J2a1a	J2a1a	175	169
I11300	0.5X	J2a1a1	2	J2a1a	J2a1	186	179
I11300	0.5X	J2a1a1	3	J2a1a1	J2a1a	189	186
I11300	0.5X	J2a1a1	4	J2a1a1	J2a1a1	181	175
I11300	0.5X	J2a1a1	5	J2a1a1	J2a1a1	182	174
I7645	0.5X	R7b2	1	F	F	105	102
I7645	0.5X	R7b2	2	HV0d	HV0d	108	103
I7645	0.5X	R7b2	3	HV0d	HV0d	103	96
I7645	0.5X	R7b2	4	F	F	114	107
I7645	0.5X	R7b2	5	HV0d	HV0d	110	105
I7646	0.5X	H1e1c	1	H1bi	H1bi	157	147
I7646	0.5X	H1e1c	2	H1e	H1e	139	133
I7646	0.5X	H1e1c	3	H58	H58	152	141
I7646	0.5X	H1e1c	4	H1e1c	H1e1c	167	162
I7646	0.5X	H1e1c	5	H1	H1	148	139
I8132	0.5X	D1a2	1	H1	H1	102	97
I8132	0.5X	D1a2	2	H1	H1	85	79
I8132	0.5X	D1a2	3	H1aq	H1aq	88	87
I8132	0.5X	D1a2	4	H1aq	H1aq	84	77
I8132	0.5X	D1a2	5	H1aq	H1aq	85	80
I8569	0.5X	H1ah	1	H1ah	H1ah	130	126
I8569	0.5X	H1ah	2	H11a6	H11a6	155	151
I8569	0.5X	H1ah	3	H3r	H3r	140	133
I8569	0.5X	H1ah	4	H1ah	H1ah	130	125
I8569	0.5X	H1ah	5	H1ah	H1ah	160	158
NW54	0.5X	C4a1a1	1	C4a1a	C4a1a	92	82
NW54	0.5X	C4a1a1	2	C4a1a	C4a1a	97	84
NW54	0.5X	C4a1a1	3	C4a1a1	C4a1a1	93	86
NW54	0.5X	C4a1a1	4	C4a1	C4a1a	102	86
NW54	0.5X	C4a1a1	5	C4a1	C4a1a1	115	94
STR266b	0.5X	J1c5	1	J1c5	J1c5	84	76
STR266b	0.5X	J1c5	2	J1c	J1c	91	80
STR266b	0.5X	J1c5	3	J1c2a3	J1c2a3	78	70
STR266b	0.5X	J1c5	4	J1c1b1	J1c1b1	85	74
STR266b	0.5X	J1c5	5	J1d	J1c	100	91
STR393b	0.5X	H5a1	1	H5a1	H5a1	121	104
STR393b	0.5X	H5a1	2	H1bc	B4a1a1u	111	93
STR393b	0.5X	H5a1	3	H5a1p	H5a1p	101	80
STR393b	0.5X	H5a1	4	H1ac	H1ac	98	78
STR393b	0.5X	H5a1	5	H1ab	H1ab	116	98
STR486	0.5X	T2b	1	T2	T2	90	78
STR486	0.5X	T2b	2	T2	T2	96	81
STR486	0.5X	T2b	3	T2	T2	96	75
STR486	0.5X	T2b	4	T2b	T2b	103	89

Continued on next page

Sample	Rate	Full_Cov_Pred	Replicate	HG_corr	HG_uncorr	Reads_corr	Reads_uncorr
STR486	0.5X	T2b	5	T2b	T2b	100	90
STR491	0.5X	T2b	1	T2b33	T2b33	90	75
STR491	0.5X	T2b	2	T2b	T2b	86	73
STR491	0.5X	T2b	3	T2b	T2b	77	67
STR491	0.5X	T2b	4	T2b	T2	109	91
STR491	0.5X	T2b	5	T2b	T2b	88	72
Vim2b	0.5X	H7	1	H27d	H41	96	81
Vim2b	0.5X	H7	2	H56a	H2a	98	89
Vim2b	0.5X	H7	3	H5a1c	H8c	113	104
Vim2b	0.5X	H7	4	HV6a	HV6a	95	85
Vim2b	0.5X	H7	5	H30a	H30a	103	92
AED204	1.0X	X2b11	1	X2b6	X2b6	199	175
AED204	1.0X	X2b11	2	X2b	X2b	221	191
AED204	1.0X	X2b11	3	X2b3	X2b3	214	178
AED204	1.0X	X2b11	4	X2b	X2b	179	149
AED204	1.0X	X2b11	5	X2b	X2b	212	187
AED92b	1.0X	U4a1	1	U4a1	U4a1e	248	205
AED92b	1.0X	U4a1	2	U4a1d	U4a1	221	185
AED92b	1.0X	U4a1	3	U4a1	U4a1	240	205
AED92b	1.0X	U4a1	4	U4a1	U4a1	259	227
AED92b	1.0X	U4a1	5	U4a1a	U4a1	246	207
Alh10	1.0X	I1	1	I1	I1	188	165
Alh10	1.0X	I1	2	I1d	I1d	185	160
Alh10	1.0X	I1	3	I1	I1	192	162
Alh10	1.0X	I1	4	I2e	I1	190	173
Alh10	1.0X	I1	5	I1	I1	183	166
DA100	1.0X	C4b1	1	C4b1	C4b1	199	183
DA100	1.0X	C4b1	2	C4b	C4b	177	161
DA100	1.0X	C4b1	3	C4b1	C4b1	199	184
DA100	1.0X	C4b1	4	C4b1	C4b1	227	218
DA100	1.0X	C4b1	5	C4b1	C4b1	203	190
DA101	1.0X	U5a1b1e	1	U5a1b1e	U5a1b1e	195	184
DA101	1.0X	U5a1b1e	2	U5a1b1e	U5a1b1e	189	175
DA101	1.0X	U5a1b1e	3	U5a1b1e	U5a1b1e	176	164
DA101	1.0X	U5a1b1e	4	U5a1b1e	U5a1b1e	181	168
DA101	1.0X	U5a1b1e	5	U5a1b1c1	U5a1b1e	173	163
DA15	1.0X	C4d	1	C4e	C4e	211	185
DA15	1.0X	C4d	2	C4e	C4	217	201
DA15	1.0X	C4d	3	C4	C4	230	202
DA15	1.0X	C4d	4	C4	C4e	223	201
DA15	1.0X	C4d	5	C4e	C4e	233	204
DA171	1.0X	H2a1	1	H3r	H3r	219	204
DA171	1.0X	H2a1	2	H2a1	H2a1	213	199
DA171	1.0X	H2a1	3	H2a1d	H2a1	237	217
DA171	1.0X	H2a1	4	H2a1	H76a	226	210
DA171	1.0X	H2a1	5	H2a1	H2a1	221	198
FN2	1.0X	H3	1	H3ar	H3ar	208	183
FN2	1.0X	H3	2	H3aq	H7e	218	190
FN2	1.0X	H3	3	H7b	H3z1	246	201
FN2	1.0X	H3	4	H25	H50	210	171

Continued on next page

Sample	Rate	Full_Cov_Pred	Replicate	HG_corr	HG_uncorr	Reads_corr	Reads_uncorr
FN2	1.0X	H3	5	H66	H66	222	194
I10899	1.0X	U5b	1	U5b1	U5b1c	367	356
I10899	1.0X	U5b	2	U5b	U5b	388	375
I10899	1.0X	U5b	3	U5b1	U5b1	367	350
I10899	1.0X	U5b	4	U5b	U5b	402	394
I10899	1.0X	U5b	5	U5b1	U5b1	357	347
I11300	1.0X	J2a1a1	1	J2a1a1	J2a1a1	362	354
I11300	1.0X	J2a1a1	2	J2a1a1	J2a1a1	325	320
I11300	1.0X	J2a1a1	3	J2a1a1	J2a1a1	361	347
I11300	1.0X	J2a1a1	4	J2a1a1	J2a1a1	364	348
I11300	1.0X	J2a1a1	5	J2a1a1	J2a1a1	370	353
I7645	1.0X	R7b2	1	HV0d	HV0d	214	205
I7645	1.0X	R7b2	2	B4'5	B4'5	200	185
I7645	1.0X	R7b2	3	HV0d	HV0d	199	187
I7645	1.0X	R7b2	4	HV0d	HV0d	208	198
I7645	1.0X	R7b2	5	H10	H10	202	196
I7646	1.0X	H1e1c	1	H1e1c	H1e1c	305	289
I7646	1.0X	H1e1c	2	H1bi	H1bi	297	284
I7646	1.0X	H1e1c	3	H1bi	H1bi	287	274
I7646	1.0X	H1e1c	4	H1bi	H1bi	289	272
I7646	1.0X	H1e1c	5	H1bi	H1bi	297	283
I8132	1.0X	D1a2	1	H1aq	H1aq	186	177
I8132	1.0X	D1a2	2	H1aq	H1aq	196	186
I8132	1.0X	D1a2	3	H1aq	H1aq	180	170
I8132	1.0X	D1a2	4	H1aq	H1aq	172	164
I8132	1.0X	D1a2	5	H1aq	H1aq	186	175
I8569	1.0X	H1ah	1	H1	H1	249	240
I8569	1.0X	H1ah	2	H1	H1	292	285
I8569	1.0X	H1ah	3	H1ah	H1ah	275	267
I8569	1.0X	H1ah	4	H1ah	H1ah	254	246
I8569	1.0X	H1ah	5	H1	H1	281	271
NW54	1.0X	C4a1a1	1	C4a1a	C4a1a	234	189
NW54	1.0X	C4a1a1	2	C4a1a	C4a1a	205	166
NW54	1.0X	C4a1a1	3	C4a1a	C4a1a	195	177
NW54	1.0X	C4a1a1	4	C4a1a1	C4a1a1	238	206
NW54	1.0X	C4a1a1	5	C4a1a1	C4a1a1	221	186
STR266b	1.0X	J1c5	1	J1c	J1c	167	153
STR266b	1.0X	J1c5	2	J1c9	J1c9	161	143
STR266b	1.0X	J1c5	3	J1c4c	J1c4c	181	159
STR266b	1.0X	J1c5	4	J1c9	J1c9	173	154
STR266b	1.0X	J1c5	5	J1	J1	176	158
STR393b	1.0X	H5a1	1	H5a	H5a	232	194
STR393b	1.0X	H5a1	2	H5a1	H5a1	218	184
STR393b	1.0X	H5a1	3	H5a1	H5a1	197	169
STR393b	1.0X	H5a1	4	H5c	H5c	247	209
STR393b	1.0X	H5a1	5	H5a1	H5a1	225	179
STR486	1.0X	T2b	1	T2	T2b33	183	157
STR486	1.0X	T2b	2	T2a3	T2a3	209	183
STR486	1.0X	T2b	3	T2b	T2b	197	172
STR486	1.0X	T2b	4	T2a3	T2a3	170	152

Continued on next page

Sample	Rate	Full_Cov_Pred	Replicate	HG_corr	HG_uncorr	Reads_corr	Reads_uncorr
STR486	1.0X	T2b	5	T2b17	T2b17	207	192
STR491	1.0X	T2b	1	T2b	T2b	177	154
STR491	1.0X	T2b	2	T2b	T2b	176	145
STR491	1.0X	T2b	3	T2b	T2b	192	157
STR491	1.0X	T2b	4	T2b	T2b	187	154
STR491	1.0X	T2b	5	T2b	T2b	158	134
Vim2b	1.0X	H7	1	H1	H1	209	191
Vim2b	1.0X	H7	2	R0	R0	194	168
Vim2b	1.0X	H7	3	H53	H53	185	161
Vim2b	1.0X	H7	4	H57	H57	187	159
Vim2b	1.0X	H7	5	H102	H102	210	177
AED204	2.0X	X2b11	1	X2b1	X2b1	391	331
AED204	2.0X	X2b11	2	X2b7	X2b7	392	341
AED204	2.0X	X2b11	3	X2b13	X2b7	411	346
AED204	2.0X	X2b11	4	X2b7	X2b1	433	369
AED204	2.0X	X2b11	5	X2b3	X2b3	411	358
AED92b	2.0X	U4a1	1	U4a1a	U4a1	458	397
AED92b	2.0X	U4a1	2	U4a1	U4a1	511	436
AED92b	2.0X	U4a1	3	U4a1a	U4a1e	500	440
AED92b	2.0X	U4a1	4	U4a1	U4a1	490	427
AED92b	2.0X	U4a1	5	U4a1a	U4a1	515	439
Alh10	2.0X	I1	1	I1	I1	343	299
Alh10	2.0X	I1	2	I1	I1	363	322
Alh10	2.0X	I1	3	I1	I1	383	330
Alh10	2.0X	I1	4	I1	I1	382	331
Alh10	2.0X	I1	5	I1	I1	375	315
DA100	2.0X	C4b1	1	C4b1	C4b1	382	358
DA100	2.0X	C4b1	2	C4b1	C4b1	381	358
DA100	2.0X	C4b1	3	C4b1	C4b1	396	358
DA100	2.0X	C4b1	4	C4b1	C4b1	371	348
DA100	2.0X	C4b1	5	C4b1	C4b1	443	410
DA101	2.0X	U5a1b1e	1	U5a1b1a1	U5a1b1a1	394	363
DA101	2.0X	U5a1b1e	2	U5a1b1e	U5a1b1e	349	321
DA101	2.0X	U5a1b1e	3	U5a1b1e	U5a1b1e	405	375
DA101	2.0X	U5a1b1e	4	U5a1b1e	U5a1b1e	411	382
DA101	2.0X	U5a1b1e	5	U5a1b1e	U5a1b1e	398	372
DA15	2.0X	C4d	1	C4d	C4d	468	427
DA15	2.0X	C4d	2	C4	C4	412	358
DA15	2.0X	C4d	3	C4d	C4d	409	370
DA15	2.0X	C4d	4	C4d	C4d	475	420
DA15	2.0X	C4d	5	C4d	C4d	465	425
DA171	2.0X	H2a1	1	H2a1	H2a1	431	398
DA171	2.0X	H2a1	2	H2a1	H3i	493	449
DA171	2.0X	H2a1	3	H76a	H76a	440	397
DA171	2.0X	H2a1	4	H2a1	H2a1	453	415
DA171	2.0X	H2a1	5	H2a1	H2a1	453	413
FN2	2.0X	H3	1	H3	H3	457	401
FN2	2.0X	H3	2	H2	H2	403	348
FN2	2.0X	H3	3	H104	H58	414	357
FN2	2.0X	H3	4	H66	H66	414	361

Continued on next page

Sample	Rate	Full_Cov_Pred	Replicate	HG_corr	HG_uncorr	Reads_corr	Reads_uncorr
FN2	2.0X	H3	5	H3	H3	465	390
I10899	2.0X	U5b	1	U5b1	U5b1	731	713
I10899	2.0X	U5b	2	U5b1	U5b1	785	753
I10899	2.0X	U5b	3	U5b1	U5b1	691	657
I10899	2.0X	U5b	4	U5b1	U5b1	732	705
I10899	2.0X	U5b	5	U5b1	U5b1	729	707
I11300	2.0X	J2a1a1	1	J2a1a1	J2a1a1	694	673
I11300	2.0X	J2a1a1	2	J2a1a1	J2a1a1	690	662
I11300	2.0X	J2a1a1	3	J2a1a1	J2a1a1	708	690
I11300	2.0X	J2a1a1	4	J2a1a1	J2a1a1	699	670
I11300	2.0X	J2a1a1	5	J2a1a1	J2a1a1	696	670
I7645	2.0X	R7b2	1	F	F	74	70
I7645	2.0X	R7b2	2	B2	B2	79	74
I7645	2.0X	R7b2	3	HV0d	HV0d	79	77
I7645	2.0X	R7b2	4	H1	H1	93	90
I7645	2.0X	R7b2	5	CZ	CZ	73	69
I7646	2.0X	H1e1c	1	H1e1c	H1e1c	549	520
I7646	2.0X	H1e1c	2	H1e1c	H1e1c	544	513
I7646	2.0X	H1e1c	3	H1e1c	H1e1c	547	513
I7646	2.0X	H1e1c	4	H1bi	H1bi	555	530
I7646	2.0X	H1e1c	5	H1e1c	H1bi	562	534
I8132	2.0X	D1a2	1	H1aq	H1aq	89	84
I8132	2.0X	D1a2	2	H1aq	H1aq	105	99
I8132	2.0X	D1a2	3	H1aq	H1aq	97	91
I8132	2.0X	D1a2	4	H1aq	H1aq	103	97
I8132	2.0X	D1a2	5	H1	H1	105	100
I8569	2.0X	H1ah	1	H1ah	H1ah	520	503
I8569	2.0X	H1ah	2	H1ah	H1ah	522	502
I8569	2.0X	H1ah	3	H1ah	H1ah	532	514
I8569	2.0X	H1ah	4	H1ah	H1ah	525	508
I8569	2.0X	H1ah	5	H1ah	H1ah	538	527
NW54	2.0X	C4a1a1	1	C4a1a1	C4a1a1	456	379
NW54	2.0X	C4a1a1	2	C4a1a1	C4a1a1	445	378
NW54	2.0X	C4a1a1	3	C4a1a	C4a1a	452	385
NW54	2.0X	C4a1a1	4	C4a1a1	C4a1a1	419	363
NW54	2.0X	C4a1a1	5	C4a1a1	C4a1a1	417	364
STR266b	2.0X	J1c5	1	J1c3g	J1c3g	355	311
STR266b	2.0X	J1c5	2	J1c	J1c	400	355
STR266b	2.0X	J1c5	3	J1c	J1c	356	315
STR266b	2.0X	J1c5	4	J1c5	J1c5	347	310
STR266b	2.0X	J1c5	5	J1c	J1c	363	319
STR393b	2.0X	H5a1	1	H5v	H5a	450	384
STR393b	2.0X	H5a1	2	H5a1	H5a1	430	359
STR393b	2.0X	H5a1	3	H5a1	H5a1	428	352
STR393b	2.0X	H5a1	4	H5a1	H5a1	465	385
STR393b	2.0X	H5a1	5	H5a1	H5a8	428	370
STR486	2.0X	T2b	1	T2b	T2	387	334
STR486	2.0X	T2b	2	T2b	T2b	387	343
STR486	2.0X	T2b	3	T2i	T2i	374	316
STR486	2.0X	T2b	4	T2b	T2b	388	336

Continued on next page

Sample	Rate	Full_Cov_Pred	Replicate	HG_corr	HG_uncorr	Reads_corr	Reads_uncorr
STR486	2.0X	T2b	5	T2b	T2b	404	353
STR491	2.0X	T2b	1	T2b	T2b	346	302
STR491	2.0X	T2b	2	T2b	T2b	333	292
STR491	2.0X	T2b	3	T2b	T2b	364	299
STR491	2.0X	T2b	4	T2b	T2b	377	326
STR491	2.0X	T2b	5	T2b	T2b	374	313
Vim2b	2.0X	H7	1	R0	R0	404	360
Vim2b	2.0X	H7	2	H105	H105	395	356
Vim2b	2.0X	H7	3	H7	H7	401	354
Vim2b	2.0X	H7	4	H3am	H3am	381	334
Vim2b	2.0X	H7	5	HV	HV	384	335

G.2 euka Experiment

G.2.1 Table of Detected Reads Per Taxon

j	Arc *tiinae*	Bov *idae*	Caly *tratae*	Colu *broidea*	Cryp *rhynchinae*	Lamp *ridae*	Lyco *soidea*	Mus	Myo *tis*	Rat *tus*	Urs *idae*	Xeri *inae*
0.0	1346	157	4300	891	185	2518	506	216	351	455	1241	152
0.1	1343	148	4293	876	185	2516	506	212	347	445	1209	148
0.2	1342	145	4271	875	185	2511	505	208	344	441	1203	147
0.3	1341	143	4257	866	185	2508	505	205	337	434	1193	146
0.4	1340	141	4247	860	185	2499	503	205	331	430	1179	144
0.5	1331	140	4222	855	184	2489	502	203	326	426	1173	142
0.6	1326	138	4203	851	183	2485	501	199	326	422	1167	142
0.7	1323	134	4195	842	182	2476	501	197	324	416	1153	142
0.8	1316	132	4178	826	181	2460	499	191	323	410	1132	142
0.9	1302	131	4141	817	178	2434	498	188	319	409	1125	142
1.0	1237	132	3980	813	177	2380	495	185	316	409	1114	143
Unc	1237	132	3980	813	177	2380	495	185	316	409	1113	143

Table 9: Reads Detected per Taxon on the euka experiment simulated metagenomic environmental aDNA sample with SAFARI as aligner at varying posterior threshold values. Note that at posterior threshold of 1.0 SAFARI and giraffe are identical. As the posterior threshold lowers more deaminated seeds are rescued and aligned, leading to an increase in detected reads across the board.

G.2.2 Detected Reads Per Taxon by SAFARI Posterior Threshold

Below (Figure 5) is simply a pictorial representation of the above table, showing how number of detected reads depends on the user-defined posterior threshold for the SAFARI RYmer seed filter. The horizontal dashed line at $y = 100$ shows the minimum number of reads required by euka to consider a taxon as detected in the sample.

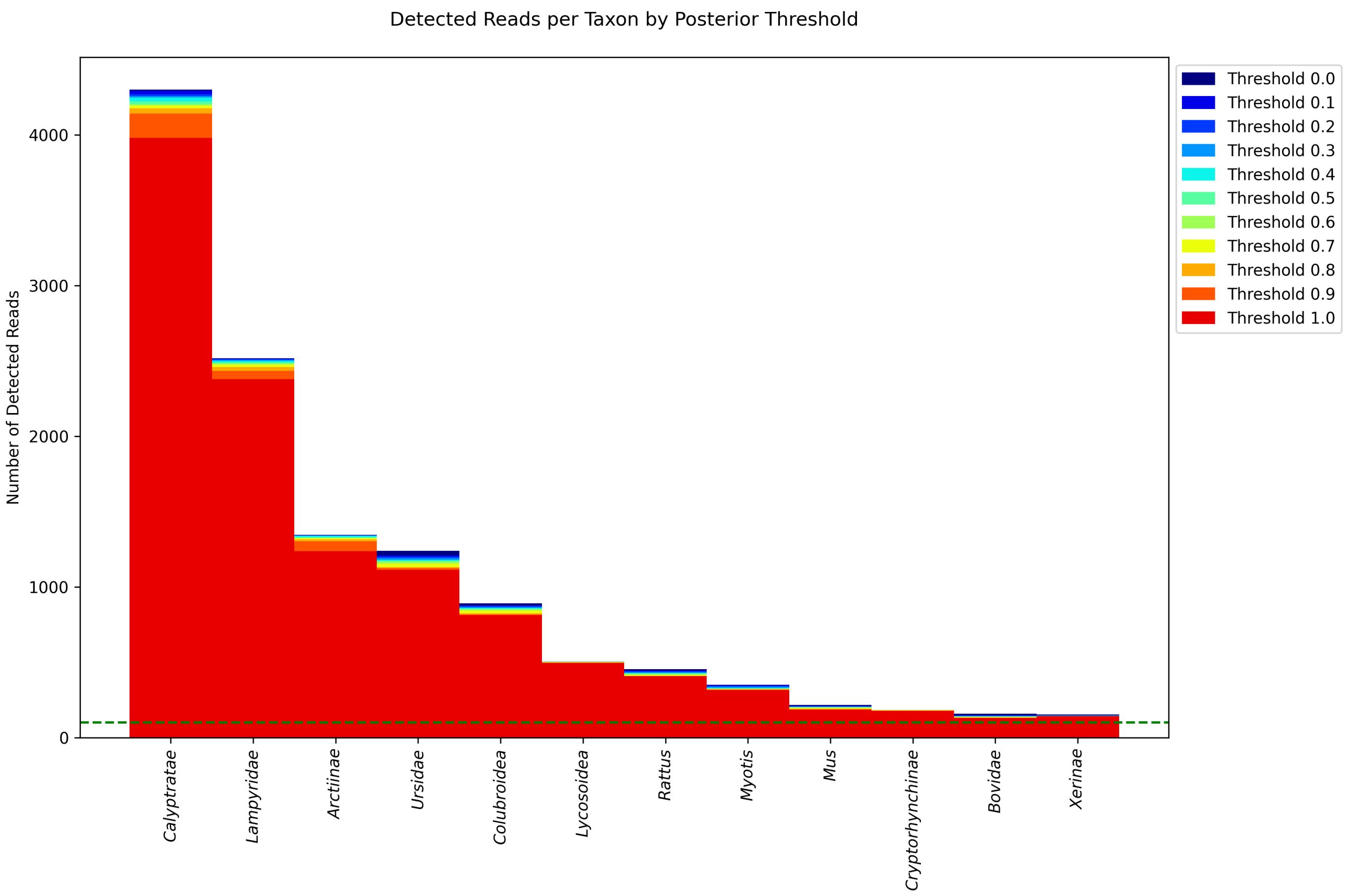

Figure 5: Number of detected reads per taxon as a function of the user-defined posterior threshold (flag `-j`) for the `SAFARI` RYmer seed filter. As the threshold decreases, more reads per taxon are detected. At a threshold of 1.0 the number of reads per taxon is identical to the results from the standard `vg giraffe`, as expected. The dashed horizontal line indicates the minimum number of fragments required to consider a taxon detected.

G.3 Linear Alignment Experiment

G.3.1 Selected Alignment Statistics

Table 10: Raw counts of mapped reads by provenance in the surjected linear alignment experiment for the dataset of *in silico* simulations following the empirically-derived fragment length distribution from Vindija Cave, Croatia under high damage rates. $NuMT$ is the number of mapped NuMT reads. For readability, only samples for a single subsampling rate (0.9) are shown. Counts are reported as aligned/total. Note that $k = 29, w = 11$ are the default parameters.

k	w	Bacterial		NuMTs		Mitochondrial	
		SAF	gir	SAF	gir	SAF	gir
10	2	51/90235	36/90235	89/360	88/360	269527/270163	269425/270163
10	5	14/90235	10/90235	83/360	83/360	269045/270163	268908/270163
10	7	8/90235	7/90235	81/360	81/360	268319/270163	268130/270163
10	8	7/90235	6/90235	79/360	79/360	267792/270163	267589/270163
10	10	7/90235	6/90235	72/360	72/360	266372/270163	266118/270163
10	15	3/90235	3/90235	65/360	65/360	258594/270163	258273/270163
11	6	5/90235	5/90235	75/360	73/360	267889/270163	267640/270163
11	7	4/90235	4/90235	72/360	71/360	267268/270163	266991/270163
12	3	3/90235	3/90235	80/360	77/360	268526/270163	268211/270163
12	4	3/90235	3/90235	79/360	75/360	268007/270163	267703/270163
12	5	3/90235	3/90235	76/360	74/360	267348/270163	267044/270163
12	6	3/90235	3/90235	75/360	74/360	266611/270163	266274/270163
12	7	3/90235	3/90235	71/360	70/360	265709/270163	265395/270163
15	5	1/90235	1/90235	54/360	54/360	260773/270163	260175/270163
15	10	0/90235	0/90235	45/360	45/360	248752/270163	248314/270163
15	15	0/90235	0/90235	33/360	33/360	231642/270163	231284/270163
18	8	0/90235	0/90235	36/360	36/360	236890/270163	236358/270163
20	2	0/90235	0/90235	40/360	35/360	244702/270163	243564/270163
20	5	0/90235	0/90235	29/360	28/360	233688/270163	233095/270163
20	10	0/90235	0/90235	19/360	19/360	213783/270163	213541/270163
20	15	0/90235	0/90235	14/360	14/360	194248/270163	194051/270163
29	11	0/90235	0/90235	8/360	5/360	138471/270163	123588/270163
30	2	0/90235	0/90235	23/360	6/360	247983/270163	166057/270163
30	5	0/90235	0/90235	9/360	5/360	200888/270163	154073/270163
30	10	0/90235	0/90235	6/360	5/360	138022/270163	120769/270163
30	15	0/90235	0/90235	6/360	5/360	95669/270163	88174/270163
**SHRiMP**							
		0/90235		86/360		269223/270163	
**BWA-MEM**							
		0/90235		27/360		237299/270163	
**BWA aln**							
		0/90235		17/360		228817/270163	
**BWA aln (anc)**							
		0/90235		19/360		251049/270163	
**Bowtie2**							
		0/90235		15/360		206858/270163	

G.3.2 Sensitivity vs. Specificity Plots

Figures 6b and 6a show sensitivity vs. specificity of the two pangenome aligners `SAFARI` and `vg giraffe` on the Chagyrskaya Cave distribution datasets for high-damage and zero-damage samples, respectively. The indexing parameters $(k, w) = (10, 2)$ were selected to (individually) optimize median sensitivity. It is evident that sensitivity benefits from `SAFARI`'s rescue of alignments containing deaminated seeds when samples are highly damaged, at the expense of a drop in specificity.

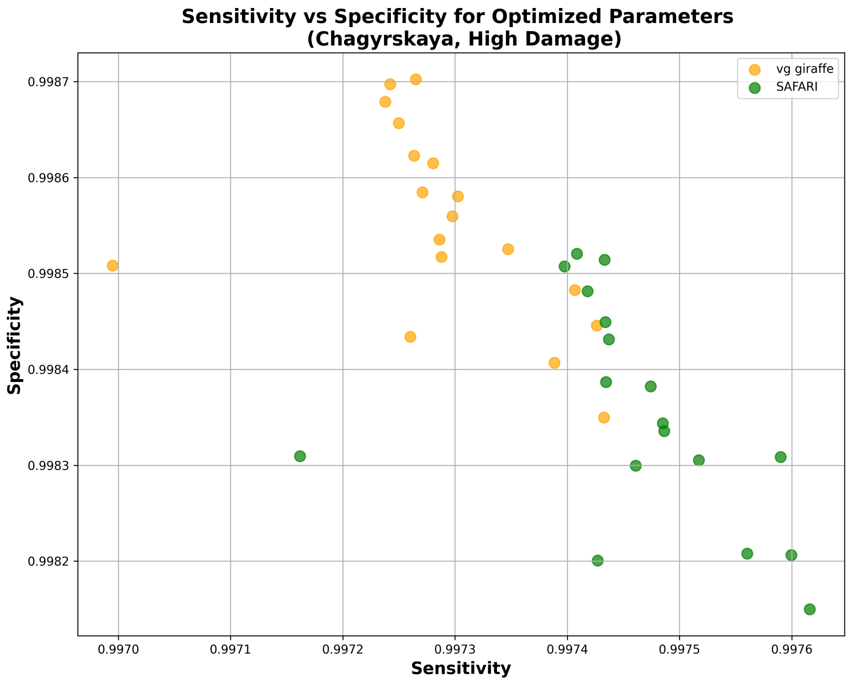

(a) High damage

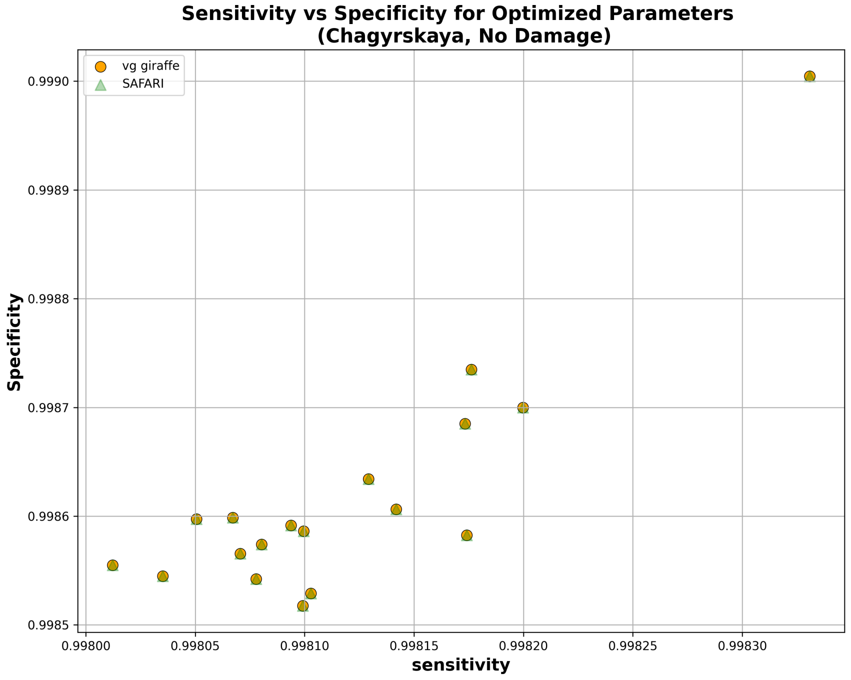

(b) Zero damage (no difference)

Figure 6: Sensitivity vs. specificity comparison of `SAFARI` and `vg giraffe` on surjected linear alignments using the empirically derived fragment length distribution from Chagyrskaya Cave, Siberia. The (k, w) indexing parameters were individually selected to optimize median sensitivity across samples, out of all tested parameter pairs. (a) Samples with high levels of damage. Here we can see that sensitivity has clearly improved with `SAFARI` thanks to the recovered alignments, at the expense of a drop in specificity. This is almost always a beneficial tradeoff in the context of aDNA analysis. (b) Samples with zero damage. As expected, there is no difference between `SAFARI` and `vg giraffe` alignments, as the resulting alignments are identical thus showing that our strategy does not generate spurious alignment in the absence of deamination.

G.3.3 Median F_1 Scores for Pangenome Aligners Using Default Indexing Parameter Values, Chagyrskaya Cave Distribution

Figure 7 shows median F_1 score for the Chagyrskaya Cave distribution dataset for each of the two pangenome aligners, stratified by damage. It is clear that by rescuing deaminated seeds, `SAFARI` improves overall alignment performance, especially on highly damaged samples.

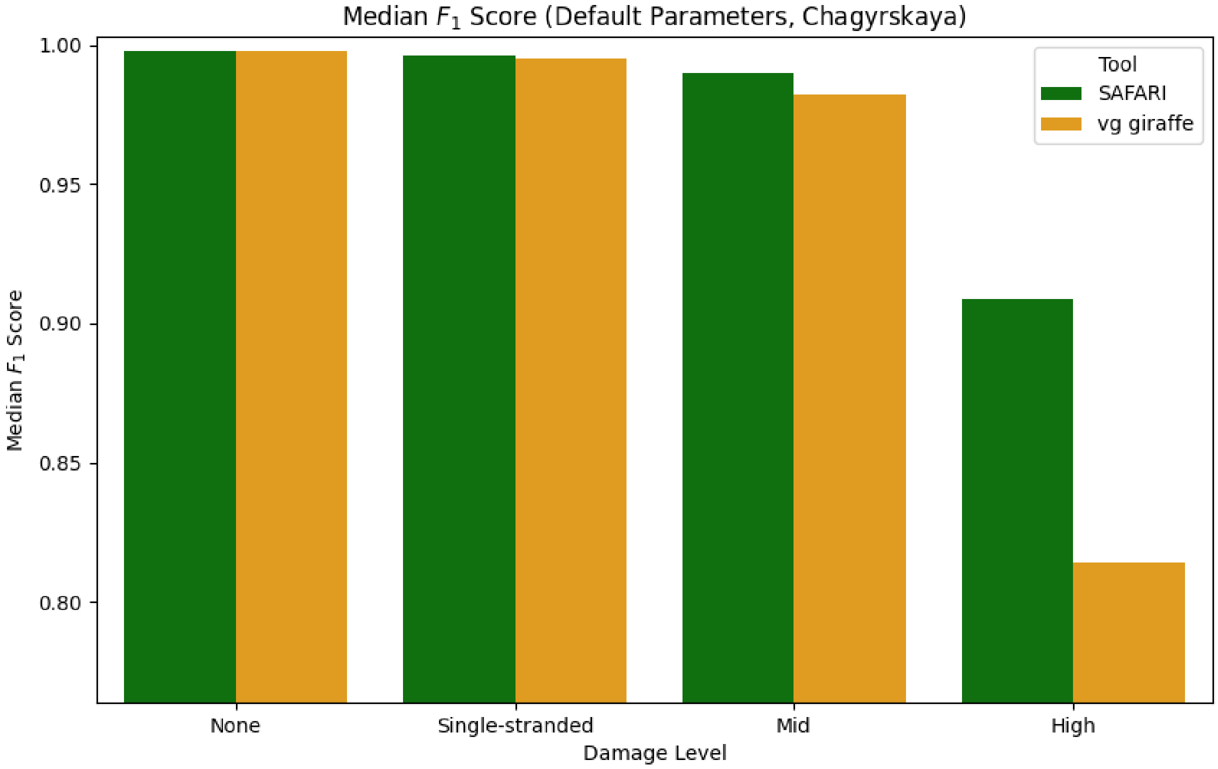

Figure 7: Median F_1 score of each of the two pangenomic alignment tools stratified by level of aDNA damage under default indexing parameters $(k, w) = (29, 11)$ for the empirically derived fragment length distribution from Chagyrskaya Cave, Croatia. The improvement in median F_1 score conferred by the deaminated seed rescue in `SAFARI` is especially clear at high levels of damage.

G.3.4 Median F_1 Scores for Pangenome Aligners Using Sensitivity-optimized Indexing Parameter Values

Figures 8a and 8b show median F_1 scores for `vg giraffe` vs. `SAFARI` on the Chagyrskaya Cave and Vindija Cave empirically-derived fragment length distribution datasets, respectively, using k, w indexing parameter values optimized for maximum sensitivity.

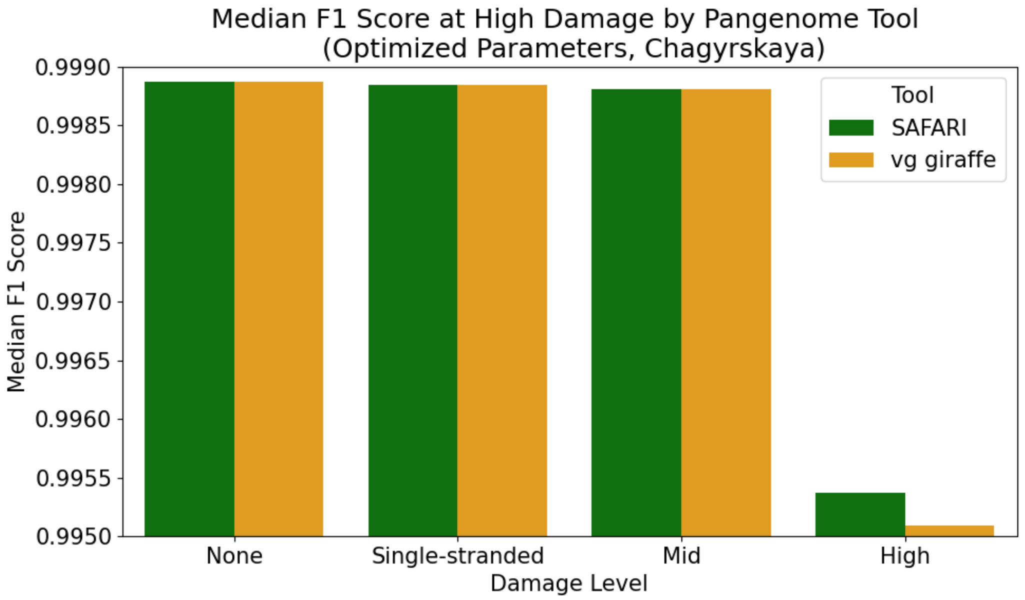

(a) Chagyrskaya Cave

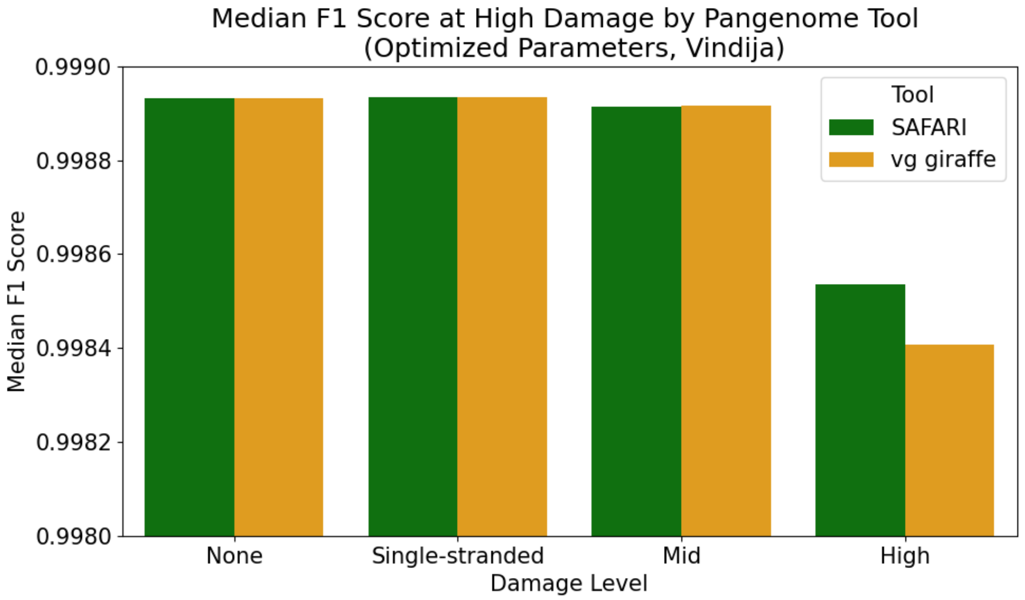

(b) Vindija Cave

Figure 8: Median F_1 scores across samples by pangenome aligner when using sensitivity-optimized k, w indexing parameter values. The optimized parameters were $(k, w) = (10, 2)$ for both tools in both cases. We show both *in silico* simulation datasets corresponding to each of the two empirically-derived fragment length distributions.

G.3.5 Differences in Median Sensitivity/Specificity Between Pangenome Aligners By Indexing Parameter Values

It is interesting to explore how altering the (k, w) indexing parameter values - that is to say the kmer size and density of the minimizer and (in the case of `SAFARI`) RYmer indexes affects the aligner's behavior in terms of where it falls along the tradeoff between sensitivity and specificity. Below (Figures 9 and 10) we plot differences in these metrics between tools by parameter values for both datasets of samples obeying empirically-derived fragment length distributions.

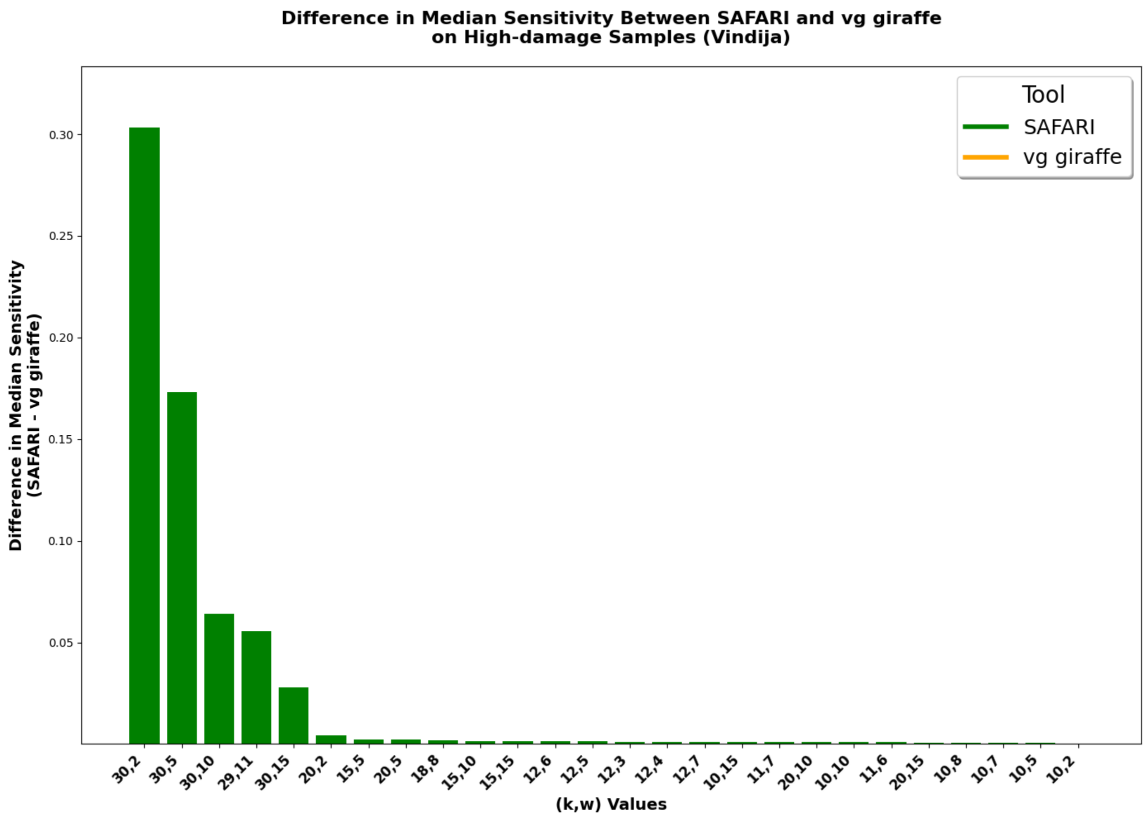

(a) Difference in median sensitivity between `SAFARI` and `vg giraffe` by indexing parameter values.

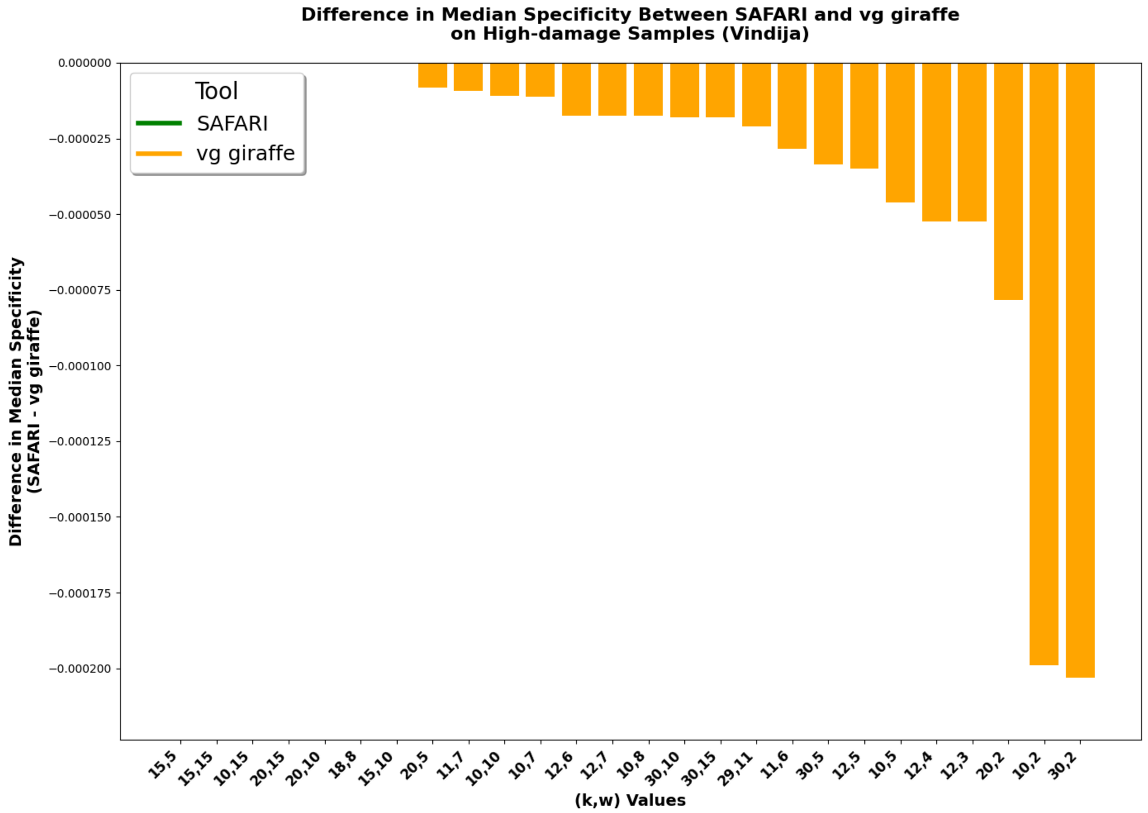

(b) Difference in median specificity between `SAFARI` and `vg giraffe` by indexing parameter values.

Figure 9: Difference in median sensitivity and median specificity between `SAFARI` and `vg giraffe` by indexing parameter values.

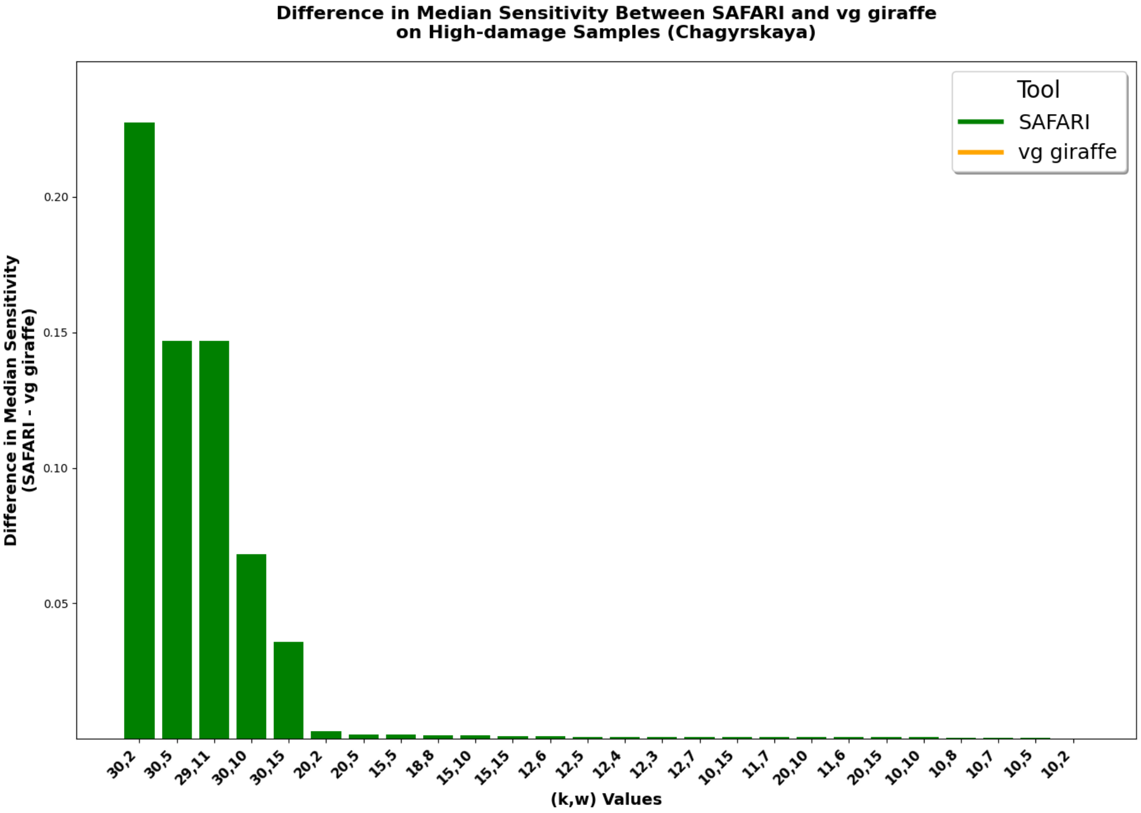

(a) Difference in median sensitivity between `SAFARI` and `vg giraffe` by indexing parameter values.

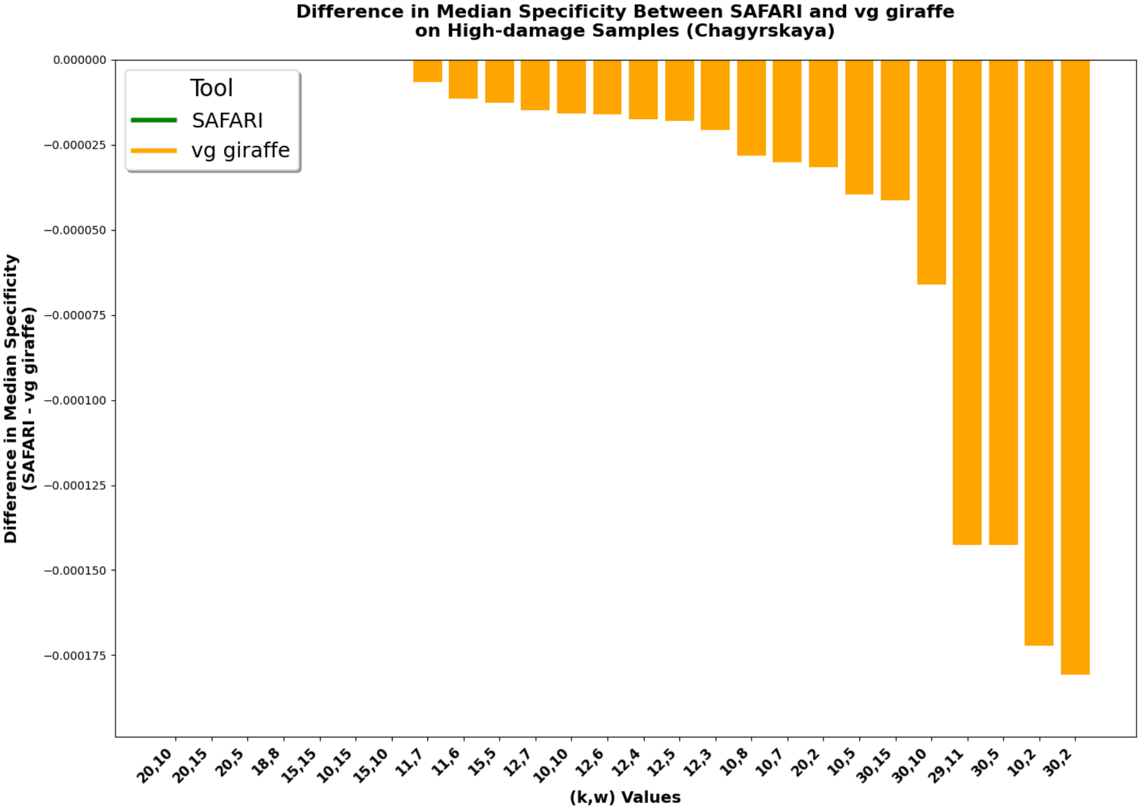

(b) Difference in median specificity between `SAFARI` and `vg giraffe` by indexing parameter values.

Figure 10: Difference in median sensitivity and median specificity between `SAFARI` and `vg giraffe` by indexing parameter values.

G.3.6 Median F_1 Scores Across All Tools Using Chagyrskaya Cave Fragment Length Distribution

Figure 11 plots median F_1 score for all tools on the Chagyrskaya Cave fragment length distribution dataset for the surjected linear alignment experiment. Similar to that of the Vindija Cave distribution we see that the two pangenome tools `vg giraffe` and `SAFARI`, in addition to `SHRiMP`, are the most robust to high levels of damage.

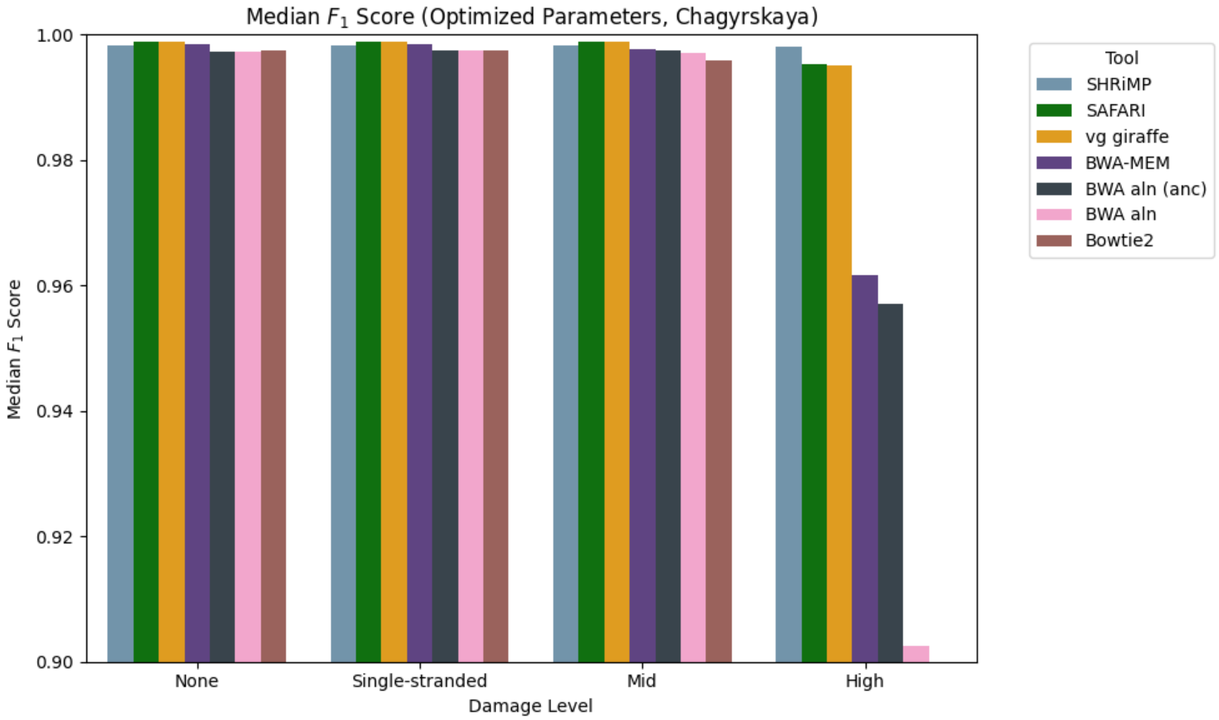

Figure 11: Median F_1 score across samples following the fragment length distribution from Chagyrskaya Cave, Siberia, stratified by level of damage. In the case of `SAFARI` and `vg giraffe` we use indexing parameter values that optimize for maximum sensitivity. We see that many tools worsen in performance precipitously once damage reaches a high level, with the two pangenome tools and `SHRiMP` being the three that are relatively robust to high damage overall.

G.3.7 Raw Counts for Pangenome Aligners on Chagyrskaya Cave Fragment Length Distribution Dataset

Reporting sensitivity, specificity, F_1, etc. is informative, but it is important to report the raw counts for the data as well. Below we provide raw counts for aligned reads by provenance (endogenous mitochondria DNA, NuMTs, or bacterial DNA) by pangenome aligner for the Chagyrskaya Cave empirical fragment length *in silico* simulation dataset.

Table 11: Raw counts of mapped reads by provenance in the surjected linear alignment experiment for the dataset of `in silico` simulations following the empirically-derived fragment length distribution from Chagyrskaya Cave, Siberia, at high-level damage rates. *Bact* stands for number of mapped bacterial reads. *NuMT* is the number of mapped NuMT reads. *Mito* is the number of correctly mapped mitochondrial reads. For readability only samples with subsampling rate 0.9 are shown.

k	w	Bact		NuMT		Mito	
		SAF	gir	SAF	gir	SAF	gir
10	2	51/90235	36/90235	96/390	95/390	269814/270545	269769/270545
10	5	14/90235	10/90235	90/390	90/390	269644/270545	269574/270545
10	7	8/90235	7/90235	86/390	85/390	269257/270545	269158/270545
10	8	7/90235	6/90235	81/390	80/390	268941/270545	268834/270545
10	10	7/90235	6/90235	77/390	77/390	268141/270545	267990/270545
10	15	3/90235	3/90235	68/390	68/390	263509/270545	263311/270545
11	6	5/90235	5/90235	85/390	84/390	268997/270545	268841/270545
11	7	4/90235	4/90235	82/390	81/390	268653/270545	268490/270545
12	3	3/90235	3/90235	91/390	89/390	269371/270545	269165/270545
12	4	3/90235	3/90235	90/390	88/390	269064/270545	268852/270545
12	5	3/90235	3/90235	84/390	83/390	268729/270545	268516/270545
12	6	3/90235	3/90235	80/390	79/390	268314/270545	268107/270545
12	7	3/90235	3/90235	76/390	75/390	267759/270545	267560/270545
15	5	1/90235	1/90235	65/390	64/390	264671/270545	264263/270545
15	10	0/90235	0/90235	49/390	49/390	257044/270545	256730/270545
15	15	0/90235	0/90235	41/390	41/390	245827/270545	245582/270545
18	8	0/90235	0/90235	35/390	35/390	249089/270545	248726/270545
20	2	0/90235	0/90235	36/390	33/390	254092/270545	253326/270545
20	5	0/90235	0/90235	30/390	30/390	246975/270545	246532/270545
20	10	0/90235	0/90235	25/390	25/390	233162/270545	232977/270545
20	15	0/90235	0/90235	21/390	21/390	218578/270545	218427/270545
29	11	0/90235	0/90235	24/390	11/390	225364/270545	185539/270545
30	2	0/90235	0/90235	30/390	12/390	256899/270545	195465/270545
30	5	0/90235	0/90235	24/390	11/390	225364/270545	185539/270545
30	10	0/90235	0/90235	15/390	9/390	179719/270545	161369/270545
30	15	0/90235	0/90235	11/390	8/390	147130/270545	137522/270545

G.3.8 Damage Rate Estimation

The below table (Table 12) shows the root mean square distance between ground truth and predicted damage rate matrices from our linear alignment experiment across all tools, both pangenomic and linear. For `SAFARI` and `vg giraffe`, the reported values are over samples with the default indexing parameters $k = 29, w = 11$. Unfortunately for reasons we were unable to determine (but which fall outside the scope of this paper) the `bam2prof` estimates failed to provide meaningful profiles for alignments generated with `Bowtie2`, instead producing profiles containing "-nan" values. This is unfortunate, but the purpose here is merely to roughly show how the pangenome tools compare to extant aligners, and discarding `Bowtie2` for this particular experiment does not negate the improvement of `SAFARI` over `vg giraffe` which is the main point.

Table 12: Median RMSE by Aligner, Stratified by Damage Matrix and Fragment Length Distribution

Fragment Length Dist	Damage Level	Aligner	Median RMSE
Chagyrskaya	None	ANC	0.000254039314235
Chagyrskaya	None	BBMap	0.000216725954602
Chagyrskaya	None	BWA aln	0.000249590309279
Chagyrskaya	None	BWA-MEM	0.000245662099981
Chagyrskaya	None	SAFARI	0.000129801168650
Chagyrskaya	None	SHRiMP	0.000240953533138
Chagyrskaya	None	vg giraffe	0.000129801168650
Chagyrskaya	Single-stranded	ANC	0.000700009013038
Chagyrskaya	Single-stranded	BBMap	0.000662971080891
Chagyrskaya	Single-stranded	BWA aln	0.000682883702541
Chagyrskaya	Single-stranded	BWA-MEM	0.000374914755779
Chagyrskaya	Single-stranded	SAFARI	0.000781440569890
Chagyrskaya	Single-stranded	SHRiMP	0.000584915345703
Chagyrskaya	Single-stranded	vg giraffe	0.000782318258900
Chagyrskaya	Mid	ANC	0.001090007922072
Chagyrskaya	Mid	BBMap	0.001061515685155
Chagyrskaya	Mid	BWA aln	0.000796128304939
Chagyrskaya	Mid	BWA-MEM	0.002429977014382
Chagyrskaya	Mid	SAFARI	0.000621920167702
Chagyrskaya	Mid	SHRiMP	0.001011073756105
Chagyrskaya	Mid	vg giraffe	0.000625510373220
Chagyrskaya	High	ANC	0.020048194493544
Chagyrskaya	High	BBMap	0.001679988895036
Chagyrskaya	High	BWA aln	0.038637014215471
Chagyrskaya	High	BWA-MEM	0.040239909990277
Chagyrskaya	High	SAFARI	0.012330074318948
Chagyrskaya	High	SHRiMP	0.002421109681100
Chagyrskaya	High	vg giraffe	0.012478861693922
Vindija	None	ANC	0.000210624637848
Vindija	None	BBMap	0.000183636136553
Vindija	None	BWA aln	0.000203554096122
Vindija	None	BWA-MEM	0.000203369211957
Vindija	None	SAFARI	0.000115545385702
Vindija	None	SHRiMP	0.000249101805604
Vindija	None	vg giraffe	0.000115545385702
Vindija	Single-stranded	ANC	0.000634061586861
Vindija	Single-stranded	BBMap	0.000614000750481
Vindija	Single-stranded	BWA aln	0.000623136335772
Vindija	Single-stranded	BWA-MEM	0.000847970960142

Continued on next page

Table 12 – continued from previous page

Fragment Length Dist	Damage Level	Aligner	Median RMSE
Vindija	Single-stranded	SAFARI	0.000561150818737
Vindija	Single-stranded	SHRiMP	0.000639254455797
Vindija	Single-stranded	vg giraffe	0.000554107280269
Vindija	Mid	ANC	0.000742789194146
Vindija	Mid	BBMap	0.001124676539555
Vindija	Mid	BWA aln	0.000596203034631
Vindija	Mid	BWA-MEM	0.003097568199287
Vindija	Mid	SAFARI	0.000976162207646
Vindija	Mid	SHRiMP	0.000717332776321
Vindija	Mid	vg giraffe	0.000979666873226
Vindija	High	ANC	0.020780023588946
Vindija	High	BBMap	0.002682043168663
Vindija	High	BWA aln	0.036673222311489
Vindija	High	BWA-MEM	0.042771379640544
Vindija	High	SAFARI	0.008651229171550
Vindija	High	SHRiMP	0.003082623667806
Vindija	High	vg giraffe	0.008684805382982

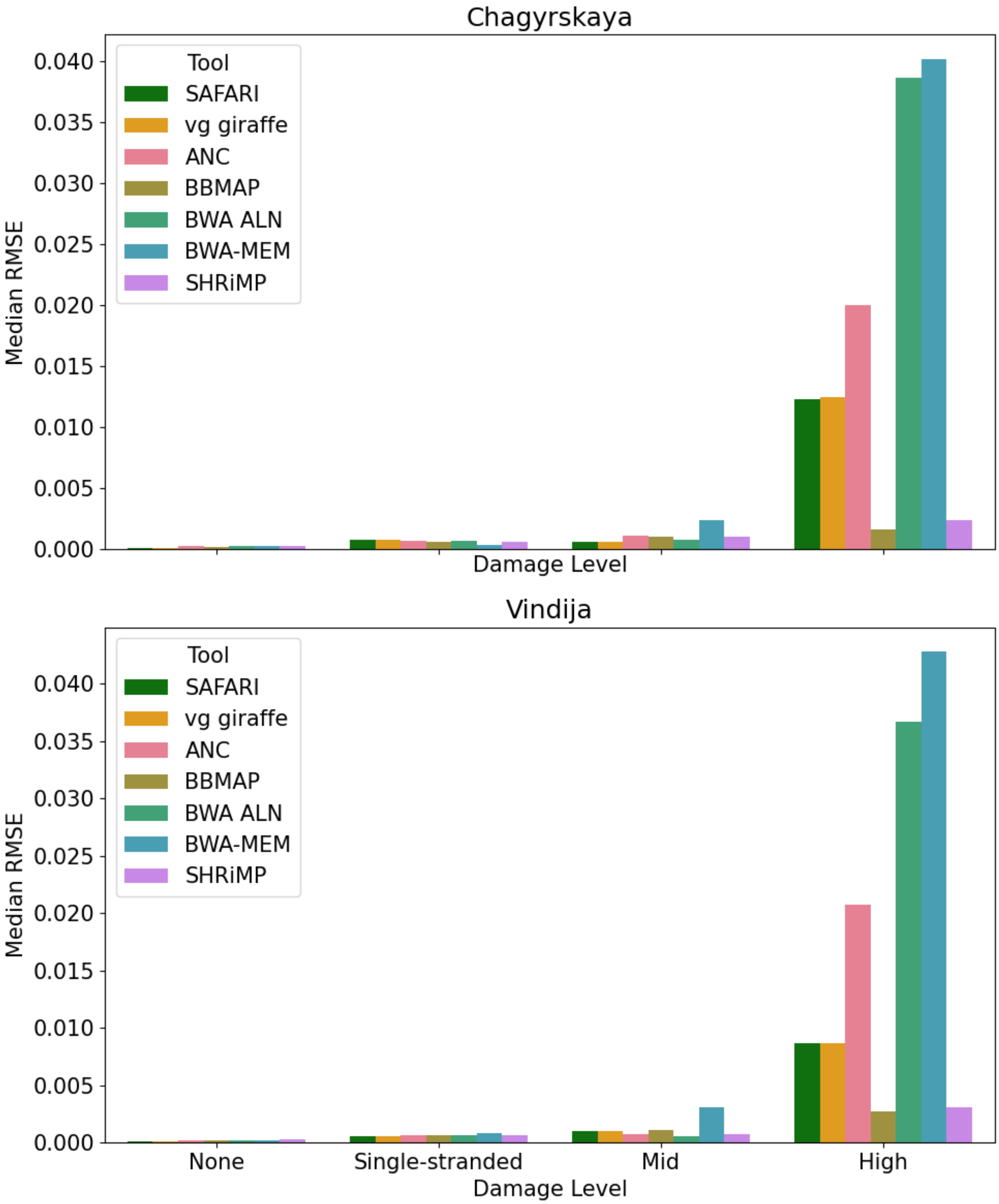

Figure 12: Median RMSE across samples for all alignment tools (both linear and pangenomic) in the surjected linear alignment experiment. [**TOP**] Samples following the empirical fragment length distribution from Chagyrskaya Cave. [**BOTTOM**] Samples following the empirical fragment length distribution from Vindija Cave. Note that the RMSE differences between `SAFARI` and `vg giraffe` are too minor to be discernible from this figure.

Damage Estimate Visualization on a High-damage Sample for Both Pangenome Aligners Although there is clear improvement, the damage estimates overall remain very similar. To illustrate how similar the damage estimates from both tools are, even for highly damaged samples, we show in Figure 13 the `bam2prof` damage rate estimates from the same high-damage input sample using both `vg giraffe` [**TOP**] and `SAFARI` [**BOTTOM**].

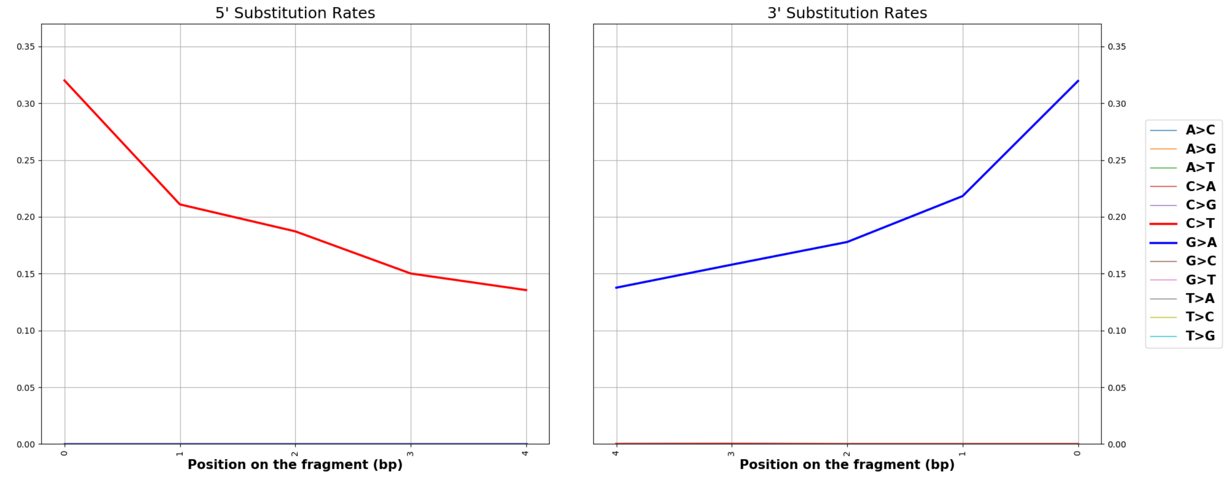

(a) `vg giraffe`

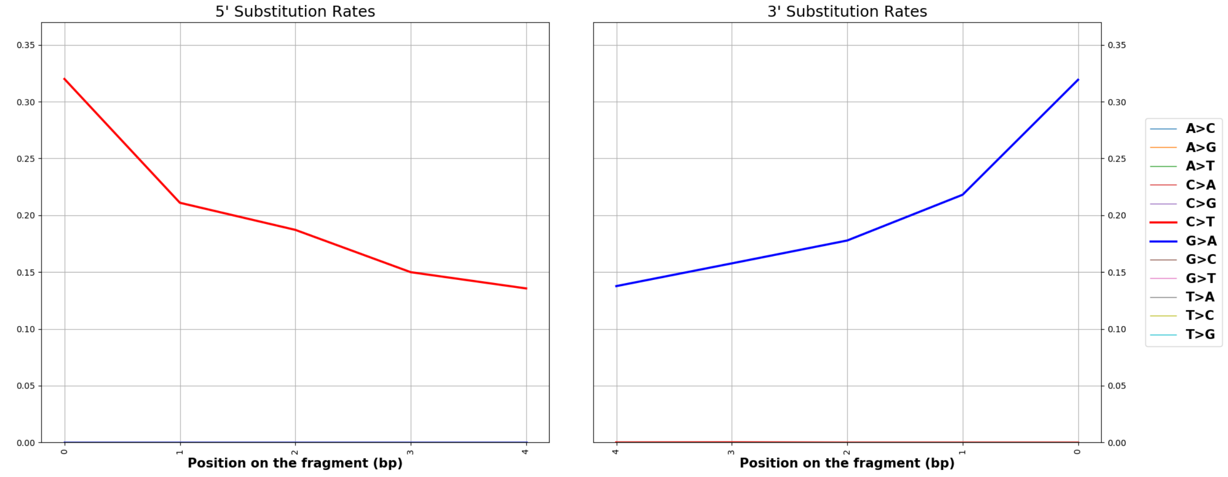

(b) `SAFARI`

Figure 13: Deamination patterns for one high-damage sample in our surjected linear alignment experiment after alignment with `vg giraffe` [**TOP**] and `SAFARI` [**BOTTOM**] obeying the empirically-derived fragment length distribution from Chagyrskaya Cave. The estimates are very nearly (though not entirely) identical.

H Pseudocode

In this section we provide some brief pseudocode to supplement our description of the RYmer-specific modification to the `vg giraffe` codebase. Of course, for full details it is best to peruse the source code itself as found in our data repository on Github.

H.1 Converting Kmer Keys to RYmer Keys

Below we demonstrate the method for converting a kmer-encoded key to its unique RYmer-encoded representation.

Algorithm 1 Kmer to RYmer Conversion

```
procedure ConvertKmerToRYmer(kmerKey, kmerLength)
    RYmerKey ← new Key()                              ▷ Initialize an empty RYmerKey
    for i ← 0 to kmerLength − 1 do
        baseType ← GetBaseType(kmerKey, i)            ▷ Determine if purine or pyrimidine
        if baseType = purine then
            RYmerKey ← RYmerKey ∘ 0                   ▷ Append 0 for purine (A or G)
        else
            RYmerKey ← RYmerKey ∘ 1                   ▷ Append 1 for pyrimidine (C or T)
    return RYmerKey
```

H.2 Filtering RYmers

Below is a thumbnail sketch of the Bayesian filter for putative Rymers described in the main manuscript. For each seed the posterior probability of genuine alignment vs. spurious alignment is computed, and putative RYmers which do not exceed the predefined threshold are discarded before clustering and extension of seeds even begin.

Algorithm 2 Filter RYmers

```
procedure FilterAndProcessRYmers
    for RYmer ∈ RYmers do
        probModel1 ← P(RYmer | model 1)
        probModel2 ← P(RYmer | model 2)
        priorModel1 ← P(model 1)
        priorModel2 ← P(model 2)
        posteriorModel1 ← (probModel1×priorModel1) / (probModel1×priorModel1+probModel2×priorModel2)
        if posteriorModel1 > threshold then
            minimizerIndexOffset ← RYmer payload
            minimizer ← minimizerIndex[minimizerIndexOffset]
            Keep minimizer
        else
            Remove RYmer
```

I Experiment Memory and Runtime

Tables 13 and 14 provide memory and runtime details for our experiments. All such data are obtained with the Linux command `/usr/bin/time -v`, except for the linear surjection experiment, which was benchmarking internally using `Snakemake`. Table 15 provides runtime information for mapping to the full hominin panmitogenome for varying (k, w) parameter pairs.

I.1 `HaploCart` and `euka`

Table 13: Comparison of runtime and memory consumption between `SAFARI` for the `HaploCart` and `euka` experiments. During these experiments some other light processes were being run on the same server node which may introduce a small amount of added noise.

		**SAFARI**	**vg giraffe**
`HaploCart`	User time (s)	10897.03	10453.30
	System time (s)	1115.41	1000.11
	Wall clock time (h:mm:ss)	18:39.25	8:14.21
	Maximum resident set size (Kb)	1879608	1335592
`euka`	User time (s)	18.49	19.64
	System time (s)	23.37	29.70
	Wall clock time (h:mm:ss)	1:06.55	0:55.93
	Maximum resident set size (Kb)	46053840	46054324

I.2 Direct Comparison (`SAFARI` vs. `vg giraffe`)

Table 14: Comparison of key performance metrics between `SAFARI` and `vg giraffe` as measured by `Snakemake` internal benchmarks.

Metric	`SAFARI`	`vg giraffe`
s	165.446	**156.658**
max RSS	615.659	**359.881**
Mean load	144.140	**137.505**

I.3 Runtime as a Function of Minimizer/RYmer Index Values

Table 15: Runtime for `SAFARI` Mapping to the Full Hominin Panmitogenome at Varying Minimizer/Rymer Index Parameters

k	w	**User Time (s)**	**System Time (s)**	**Elapsed Time (h:mm:ss)**
10	2	4433.88	23.76	1:45.21
12	3	3438.49	20.41	1:13.80
15	5	2575.69	16.08	0:52.76
17	10	1639.84	13.65	0:47.92
20	10	1649.12	13.92	0:48.89
20	30	791.52	7.74	0:22.19
29	11	1225.73	7.61	0:23.17
29	30	625.39	3.79	0:12.29

J Experiment Data Details

J.1 Nucleotide Misincorporation Matrices for Simulated Data

Nucleotide misincorporation matrices for high rates of damage are taken from [8]. Matrices for medium rates of damage are taken from [24]. Matrices for single-stranded rates of damage are taken from [22]. ATP2 matrices from a Denisovan sample (used for the experiments in Section D.5 and D.6) are taken from [31].

Table 16: Zero damage nucleotide misincorporation rate matrix for the 3' end.

	A			C			G			T		
	→C	→G	→T	→A	→G	→T	→A	→C	→T	→A	→C	→G
-4	0	0	0	0	0	0	0	0	0	0	0	0
-3	0	0	0	0	0	0	0	0	0	0	0	0
-2	0	0	0	0	0	0	0	0	0	0	0	0
-1	0	0	0	0	0	0	0	0	0	0	0	0
0	0	0	0	0	0	0	0	0	0	0	0	0

Table 17: Zero damage nucleotide misincorporation rate matrix for the 5' end.

	A			C			G			T		
	→C	→G	→T	→A	→G	→T	→A	→C	→T	→A	→C	→G
0	0	0	0	0	0	0	0	0	0	0	0	0
1	0	0	0	0	0	0	0	0	0	0	0	0
2	0	0	0	0	0	0	0	0	0	0	0	0
3	0	0	0	0	0	0	0	0	0	0	0	0
4	0	0	0	0	0	0	0	0	0	0	0	0

Table 18: Double-stranded medium damage nucleotide misincorporation rate matrix for the 3' end.

	A			C			G			T		
	→C	→G	→T	→A	→G	→T	→A	→C	→T	→A	→C	→G
-4	0	0	0	0	0	0	0.0160588	0	0	0	0	0
-3	0	0	0	0	0	0	0.0171117	0	0	0	0	0
-2	0	0	0	0	0	0	0.0260759	0	0	0	0	0
-1	0	0	0	0	0	0	0.0483092	0	0	0	0	0
0	0	0	0	0	0	0	0.13679	0	0	0	0	0

Table 19: Double-stranded medium damage nucleotide misincorporation rate matrix for the 5' end.

	A			C			G			T		
	→C	→G	→T	→A	→G	→T	→A	→C	→T	→A	→C	→G
0	0	0	0	0	0	0.173982	0	0	0	0	0	0
1	0	0	0	0	0	0.0703105	0	0	0	0	0	0
2	0	0	0	0	0	0.0259287	0	0	0	0	0	0
3	0	0	0	0	0	0.0145445	0	0	0	0	0	0
4	0	0	0	0	0	0.0135411	0	0	0	0	0	0

Table 20: Double-stranded high damage nucleotide misincorporation rate matrix for the 3' end.

	A			C			G			T		
	→C	→G	→T	→A	→G	→T	→A	→C	→T	→A	→C	→G
-4	0	0	0	0	0	0	0.146352	0	0	0	0	0
-3	0	0	0	0	0	0	0.164419	0	0	0	0	0
-2	0	0	0	0	0	0	0.188599	0	0	0	0	0
-1	0	0	0	0	0	0	0.223405	0	0	0	0	0
0	0	0	0	0	0	0	0.32891	0	0	0	0	0

Table 21: Double-stranded high damage nucleotide misincorporation rate matrix for the 5' end.

	A			C			G			T		
	→C	→G	→T	→A	→G	→T	→A	→C	→T	→A	→C	→G
0	0	0	0	0	0	0.329405	0	0	0	0	0	0
1	0	0	0	0	0	0.221745	0	0	0	0	0	0
2	0	0	0	0	0	0.187678	0	0	0	0	0	0
3	0	0	0	0	0	0.161196	0	0	0	0	0	0
4	0	0	0	0	0	0.144011	0	0	0	0	0	0

Table 22: Single-stranded low damage nucleotide misincorporation rate matrix for the 3' end.

	A			C			G			T		
	→C	→G	→T	→A	→G	→T	→A	→C	→T	→A	→C	→G
-4	0	0	0	0	0	0.00242025	0	0	0	0	0	0
-3	0	0	0	0	0	0.0035038	0	0	0	0	0	0
-2	0	0	0	0	0	0.00400941	0	0	0	0	0	0
-1	0	0	0	0	0	0.0277661	0	0	0	0	0	0
0	0	0	0	0	0	0.074432	0	0	0	0	0	0

Table 23: Single-stranded low damage nucleotide misincorporation rate matrix for the 5' end.

	A			C			G			T		
	→C	→G	→T	→A	→G	→T	→A	→C	→T	→A	→C	→G
0	0	0	0	0	0	0.0329203	0	0	0	0	0	0
1	0	0	0	0	0	0.00318909	0	0	0	0	0	0
2	0	0	0	0	0	0.00279628	0	0	0	0	0	0
3	0	0	0	0	0	0.00222379	0	0	0	0	0	0
4	0	0	0	0	0	0.00274437	0	0	0	0	0	0

Table 24: ATP2 damage nucleotide misincorporation rate matrix for the 3' end.

	A			C			G			T		
	→C	→G	→T	→A	→G	→T	→A	→C	→T	→A	→C	→G
-4	0	0	0	0	0	0	0.32891	0	0	0	0	0
-3	0	0	0	0	0	0	0.223405	0	0	0	0	0
-2	0	0	0	0	0	0	0.188599	0	0	0	0	0
-1	0	0	0	0	0	0	0.164419	0	0	0	0	0
0	0	0	0	0	0	0	0.146352	0	0	0	0	0

Table 25: ATP2 damage nucleotide misincorporation rate matrix for the 5' end.

	A			C			G			T		
	→C	→G	→T	→A	→G	→T	→A	→C	→T	→A	→C	→G
0	0	0	0	0	0	0.329405	0	0	0	0	0	0
1	0	0	0	0	0	0.221745	0	0	0	0	0	0
2	0	0	0	0	0	0.187678	0	0	0	0	0	0
3	0	0	0	0	0	0.161196	0	0	0	0	0	0
4	0	0	0	0	0	0.144011	0	0	0	0	0	0

J.2 Downsampled Ancient BAM Files for HaploCart Experiment

The forty BAM files used in the HaploCart experiment are publicly available from the European Nucleotide Archive (ENA)[15]. Accession numbers are provided in [29], S1 file Section 2.7.

J.3 Simulated Metagenomic Environment for euka Experiment

We generated a simplified metagenomic environment by modifying the published Snakefile emulating a Late Pleistocene cave environment from Mexico (https://github.com/nicolaavogel/eukaPaperData/tree/master/CreateSimulatedEnv/Snakefile_cave_env). A detailed discussion of the curated and processed data for this environment is provided in [33], Supplementary Material Section 8. For the purposes of our SAFARI benchmarking experiment we somewhat simplified this environment. The modified Snakefile can be found at https://github.com/JoshuaDanielRubin/SAFARI_paper_data/blob/main/cave_env_Snakefile.

In our environment we included three eukaryotic taxa, namely *Ursidae* (bears), *Sciuridae* (squirrels), and *Falconiformes* (falcons and related birds). 5000 fragments of each taxon were present in the environment. Also present were 40000 viral fragments, 80000 archaeal fragments, 80000 bacterial fragments, 10000 plantal fragments, and 60000 fungal fragments. In addition 25000 nuclear fragments from each of the present eukaryotic taxa were incorporated. aDNA damage was simulated using the deamSim subcommand of gargammel using the high damage rate matrices provided in Section J.1[28].

References

[1] Richard M Andrews et al. "Reanalysis and Revision of the Cambridge Reference Sequence for Human Mitochondrial DNA". In: *Nature Genetics* 23.2 (1999), pp. 147–147.

[2] Vladimir Bajić, Vanessa Hava Schulmann, and Katja Nowick. "mtDNA" Nomenclutter" and its Consequences on the Interpretation of Genetic Data". In: *bioRxiv* (2023), pp. 2023–11.

[3] Dennis A Benson et al. "GenBank". In: *Nucleic Acids Research* 41.D1 (2012), pp. D36–D42.

[4] Brian Bushnell. *BBMap: A Fast, Accurate, Splice-Aware Aligner.* Tech. rep. Lawrence Berkeley National Lab. (LBNL), Berkeley, CA (United States), 2014.

[5] Jinlyung Choi et al. "Strategies to Improve Reference Databases for Soil Microbiomes". In: *The ISME Journal* 11.4 (2017), pp. 829–834.

[6] Arne Dur, Nicole Huber, and Walther Parson. "Fine-Tuning Phylogenetic Alignment and Haplogrouping of mtDNA Sequences". In: *International Journal of Molecular Sciences* 22.11 (2021), p. 5747.

[7] Erik Garrison et al. "Building Pangenome Graphs". In: *bioRxiv* (2023), pp. 2023–04.

[8] Torsten Günther et al. "Ancient Genomes Link Early Farmers from Atapuerca in Spain to Modern-Day Basques". In: *Proceedings of the National Academy of Sciences* 112.38 (2015), pp. 11917–11922.

[9] Weichun Huang et al. "ART: A Next-Generation Sequencing Read Simulator". In: *Bioinformatics* 28.4 (2012), pp. 593–594.

[10] Joshua D Kapp, Richard E Green, and Beth Shapiro. "A Fast and Efficient Single-Stranded Genomic Library Preparation Method Optimized for Ancient DNA". In: *Journal of Heredity* 112.3 (2021), pp. 241–249.

[11] Jeffrey M Kidd et al. "Mapping and Sequencing of Structural Variation from Eight Human Genomes". In: *Nature* 453.7191 (2008), pp. 56–64.

[12] Motoo Kimura. "Estimation of Evolutionary Distances Between Homologous Nucleotide Sequences". In: *Proceedings of the National Academy of Sciences* 78.1 (1981), pp. 454–458.

[13] Johannes Köster and Sven Rahmann. "Snakemake—a Scalable Bioinformatics Workflow Engine". In: *Bioinformatics* 28.19 (2012), pp. 2520–2522.

[14] Ben Langmead and Steven L Salzberg. "Fast gapped-read alignment with Bowtie 2". In: *Nature methods* 9.4 (2012), pp. 357–359.

[15] Rasko Leinonen et al. "The European Nucleotide Archive". In: *Nucleic Acids Research* 39.suppl_1 (2010), pp. D28–D31.

[16] Heng Li. "Aligning Sequence Reads, Clone Sequences and Assembly Contigs with BWA-MEM". In: *arXiv preprint arXiv:1303.3997* (2013).

[17] Heng Li. *seqtk: A fast and Lightweight Tool for Processing Sequences in the FASTA or FASTQ Format.* GitHub repository. 2012. URL: https://github.com/lh3/seqtk.

[18] Heng Li and Richard Durbin. "Fast and Accurate Short Read Alignment with Burrows–Wheeler Transform". In: *Bioinformatics* 25.14 (2009), pp. 1754–1760.

[19] Heng Li et al. "The Sequence Alignment/map Format and SAMtools". In: *Bioinformatics* 25.16 (2009), pp. 2078–2079.

[20] Fabrizio Mafessoni et al. "A High-coverage Neandertal Genome from Chagyrskaya Cave". In: *Proceedings of the National Academy of Sciences* 117.26 (2020), pp. 15132–15136.

[21] Rui Martiniano et al. "Removing Reference Bias and Improving Indel Calling in Ancient DNA Data Analysis by Mapping to a Sequence Variation Graph". In: *Genome Biology* 21.1 (2020), pp. 1–18.

[22] Matthias Meyer et al. "A High-Coverage Genome Sequence from an Archaic Denisovan Individual". In: *Science* 338.6104 (2012), pp. 222–226.

[23] Lam-Tung Nguyen et al. "IQ-TREE: A Fast and Effective Stochastic Algorithm for Estimating Maximum-Likelihood Phylogenies". In: *Molecular Biology and Evolution* 32.1 (2015), pp. 268–274.

[24] Iñigo Olalde et al. “Derived immune and ancestral pigmentation alleles in a 7,000-year-old Mesolithic European”. In: *Nature* 507.7491 (2014), pp. 225–228.

[25] Kay Prüfer et al. “A High-Coverage Neandertal Genome from Vindija Cave in Croatia”. In: *Science* 358.6363 (2017), pp. 655–658.

[26] Gabriel Renaud. *bam2prof*. https://github.com/grenaud/bam2prof. Accessed: 2024-07-24.

[27] Gabriel Renaud, Udo Stenzel, and Janet Kelso. “leeHom: Adaptor Trimming and Merging for Illumina Sequencing Reads”. In: *Nucleic Acids Research* 42.18 (2014), e141–e141.

[28] Gabriel Renaud et al. “gargammel: a Sequence Simulator for Ancient DNA”. In: *Bioinformatics* 33.4 (2017), pp. 577–579.

[29] Joshua Daniel Rubin et al. “HaploCart: Human mtDNA Haplogroup Classification Using a Pangenomic Reference Graph Human mtDNA Haplogroup Inference”. In: *PLOS Computational Biology* 19.6 (2023), e1011148.

[30] Stephen M Rumble et al. “SHRiMP: Accurate Mapping of Short Color-Space Reads”. In: *PLoS Computational Biology* 5.5 (2009), e1000386.

[31] Susanna Sawyer et al. “Nuclear and Mitochondrial DNA Sequences from Two Denisovan Individuals”. In: *Proceedings of the National Academy of Sciences* 112.51 (2015), pp. 15696–15700.

[32] Domenico Simone et al. “The Reference Human Nuclear Mitochondrial Sequences Compilation Validated and Implemented on the UCSC Genome Browser”. In: *BMC Genomics* 12.1 (2011), pp. 1–11.

[33] Nicola Alexandra Vogel et al. “euka: Robust Tetrapodic and Arthropodic Taxa Detection from Modern and Ancient Environmental DNA using Pangenomic Reference Graphs”. In: *Methods in Ecology and Evolution* (2023). DOI: 10.1111/2041-210X.14214. URL: https://doi.org/10.1111/2041-210X.14214.

[34] Wenhao Xu et al. “An Efficient Pipeline for Ancient DNA Mapping and Recovery of Endogenous Ancient DNA from Whole-genome Sequencing Data”. In: *Ecology and Evolution* 11.1 (2021), pp. 390–401.

[35] De-Xing Zhang and Godfrey M Hewitt. “Nuclear Integrations: Challenges for Mitochondrial DNA Markers”. In: *Trends in Ecology & Evolution* 11.6 (1996), pp. 247–251.
